# Explaining long-term outcome trajectories in social–ecological systems

**DOI:** 10.1371/journal.pone.0215230

**Published:** 2019-04-15

**Authors:** Pushpendra Rana, Daniel C. Miller

**Affiliations:** Department of Natural Resources and Environmental Sciences, University of Illinois, Urbana, IL, United States of America; Assam University, INDIA

## Abstract

Improved knowledge of long-term social and environmental trends and their drivers in coupled human and natural systems is needed to guide nature and society along a more sustainable trajectory. Here we combine common property theory and experimental impact evaluation methods to develop an approach for analyzing long-term outcome trajectories in social–ecological systems (SESs). We constructed robust counterfactual scenarios for observed vegetation outcome trajectories in the Indian Himalaya using synthetic control matching. This approach enabled us to quantify the contribution of a set of biophysical and socioeconomic factors in shaping observed outcomes. Results show the relative importance of baseline vegetation condition, governance, and demographic change in predicting long-term ecological outcomes. More generally, the findings suggest the broad potential utility of our approach to analyze long-term outcome trajectories, target new policy interventions, and assess the impacts of policies on sustainability goals in SESs across the globe.

## Introduction

Ensuring environmental sustainability while improving human well-being in the context of climate change is an increasingly critical global challenge [1,2]. To address this challenge policymakers and scholars are developing a range of new approaches to promote and assess sustainability in coupled human and natural systems [3,4]. One promising way forward is to analyze potentially favorable long-term trends and their drivers to help guide nature–society interactions toward more sustainable trajectories [5,6]. Retrospective research using rigorous ex-post analyses can help prioritize regulatory and other governance actions to further sustainability goals [7,8] and may help establish appropriate counterfactual reference scenarios to measure the performance of climate mitigation and other environmental policies against their stated goals [9]. However, understanding social and ecological sustainability requires a long time horizon, adequate data, and methods that allow outcomes to be tracked and assessed.

Common property scholarship has shed important light on a range of key factors associated with sustainable natural resource governance. Ostrom’s design principles provide a foundation to understand how common pool resources may be sustainably governed over the long term [10]. These principles have now been refined [11,12] and validated in many different contexts [3,12,13], with recent work empirically examining how they scale [14,15]. Ostrom [16] further developed a social–ecological system (SES) framework to analyze connections and feedbacks between human and natural systems to generate insights for solving critical governance challenges. This framework provides a means to understand factors and processes relating to the sustainability of forests as common property resources [3,16].

To date, however, empirical studies within the common property literature have largely relied on qualitative or cross-sectional data, often from single cases, to probe the relationships and contextual conditions associated with sustainable resource governance. This approach yields only a partial understanding of the causal efficacy of key variables and factors predictive of long-term social and ecological outcome trajectories, and it struggles to address issues of multiple and contingent correlation, noncomparability of results from different studies, and spurious correlation [17,18]. Common property research has made scant use of experimental and quasi-experimental impact evaluation techniques that are increasingly employed to assess environmental programs and policies [19,20]. However, impact evaluation studies in environmental policy have mostly focused on near-term impacts rather than longer-term outcomes [19,21].

There is now a need to bring the common property and policy impact evaluation literature together to respond to critical challenges in the sustainable governance of natural resources. For example, contextually rich common property research may provide critical questions and insights that can be formalized and tested in impact evaluation scholarship. Recent advances in impact evaluation research can provide methodological tools to rigorously examine factors that have higher relative importance in explaining observed outcome trajectories in SESs [20,22]. Here we use such an advance, the synthetic control method [23–26], to identify a set of predictive factors and their relative importance in shaping long-term outcomes. Rather than using an impact evaluation approach to test the impact of a particular intervention at a single point in time as commonly done [21], our approach enables identification of impacts at multiple points in time so as to trace long-term outcome trajectories.

The objective of the study are twofold. First, we develop and test a new approach for analyzing key factors that are predictive of long-term outcomes in SES. This approach is based on the integration of insights and methods from scholarly literatures on common property and policy impact evaluation. Second, we use the approach to identify and analyze key predictors that are structurally associated with observed long-term vegetation growth trajectories in a SES in the Indian Himalaya. This second objective entails ranking predictors associated with different levels of vegetation growth to explore heterogeneity in long-term outcome trajectories and the underlying predictors.

## Methods

**Fig 1:**
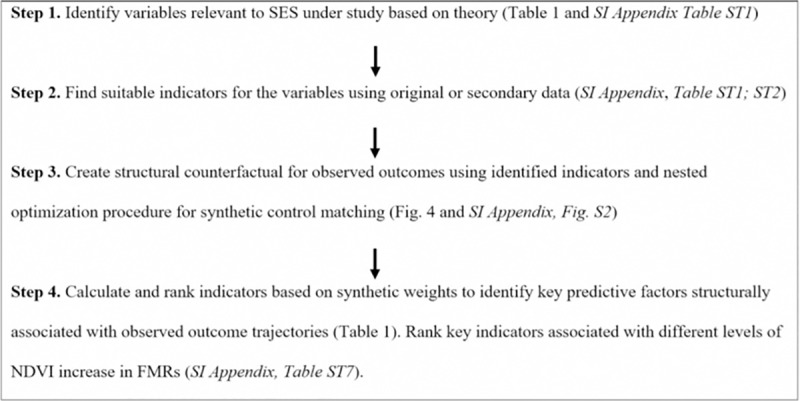
A four-step approach to identify critical factors associated with the long-term environmental outcome trajectories in social-ecological systems.

**Fig 2:**
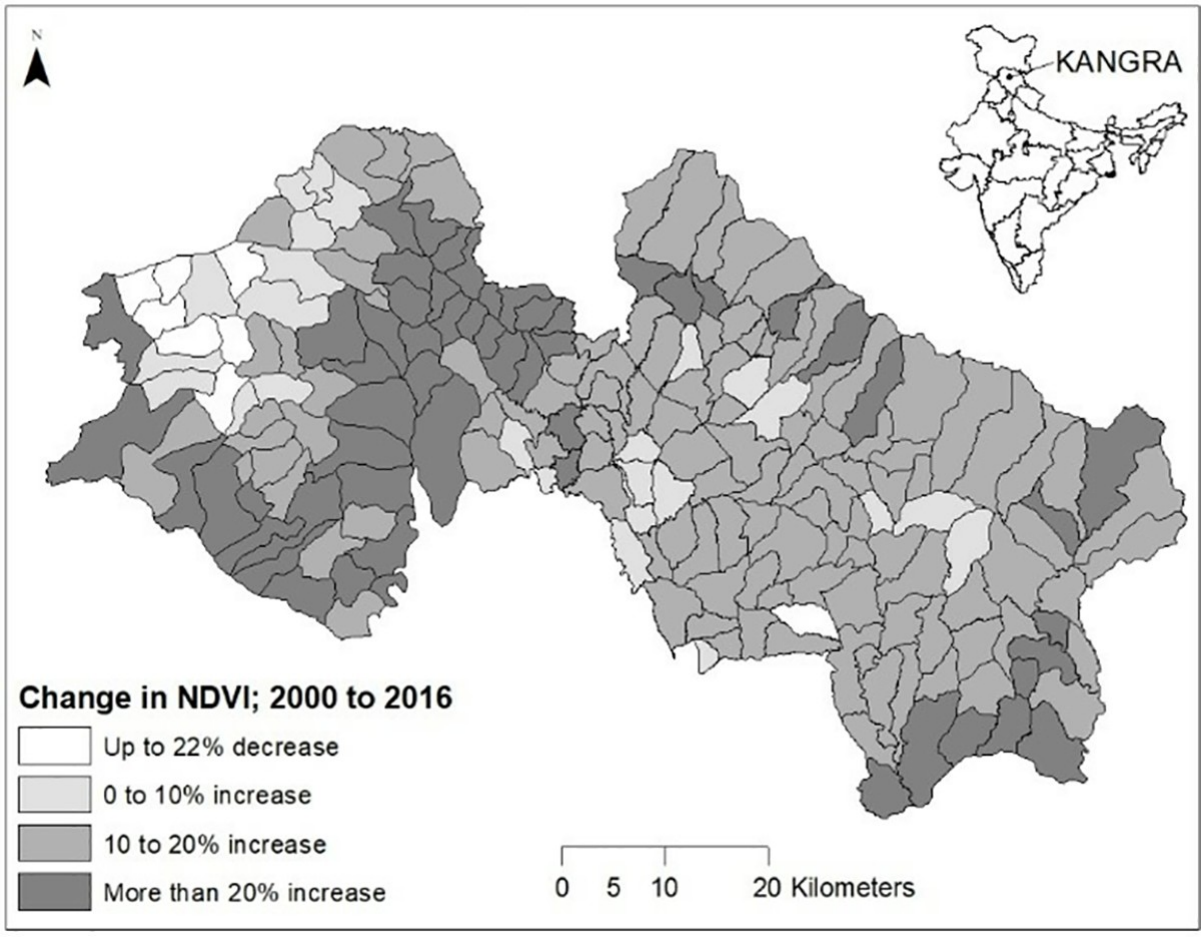
Kangra District, Himachal Pradesh, India, showing change in mean annual normalized difference vegetation index (NDVI) for the studied FMRs, 2002–2016.

Our approach for analyzing key factors predictive of long-term trends in ecological outcomes in SESs includes four steps, as described in Fig 1. The approach is based on a SES framework, which we then operationalize using a literature-based theory of change (Figure A and Tables A-C in S1 File) and publicly available data from Kangra District, Himachal Pradesh, India (Table B in S1 File). The study area (Fig 2) includes 202 Forest Management Regions (FMRs) in Kangra district. To develop a representative set of structural counterfactuals necessary to identify key factors shaping observed long-term vegetation growth trajectories in the study area (Step 3 in Fig 1) we drew a random sample of 30 out of the 202 FMRs in Kangra.

We chart the observed outcome trajectory for vegetation using a mean annual normalized difference vegetation index (NDVI) for the studied FMRs from 2002 to 2016 [27] and then use a nested optimization procedure developed for synthetic control matching (SCM) to create a structural counterfactual to observed vegetation outcome trajectories for selected FMRs based on indicators we identified (Fig 1; see below for a detailed description of the method). As the fourth and final step in our approach, we calculate and rank critically important factors associated with observed long-term ecological growth based on synthetic weights. This procedure enables testing of theories from common property literature to draw conclusions regarding the constellation of relevant variables that explain observed ecological outcome trajectories over the long term.

### Study area

We selected Kangra District in the Indian Himalaya to explore the factors and processes that explain greater potential for social–ecological sustainability. The area has been well studied, is relatively data-rich, and has experienced increased vegetation growth over the past fifteen years. Common property scholars have previously conducted intensive research on the social–ecological determinants of forest condition in Kangra District [13,28].

We examine the dynamics of vegetation growth since vegetation, including forest area, provides crucial socioeconomic and ecological benefits in the region. For example, vegetation constitutes an important livelihood base by supplying green fodder, construction timber, and fuelwood for most of the population, carbon mitigation, and species habitat [13,28]. Forest and wetland areas also provide key regulating and other ecosystem services that include maintenance of soil fertility, retention of soil moisture, and prevention of soil erosion [29].

We expect that a range of often interlinked causal factors and mechanisms will shape long-term trends in vegetation growth (Figure A and Table C in S1 File). Higher rates of literacy, for instance, may have expanded off-farm job opportunities leading to abandonment of agricultural fields and increasing vegetation growth [30]. The methodology we develop and apply to identify critical determinants of long-term trajectories in the Himalayan study context is broadly relevant and can be employed in other SESs around the world to build knowledge of factors leading to sustainable natural resource governance.

### Outcome variable (long-term NDVI trajectories)

NDVI was used as a proxy for estimating long-term vegetation growth. Consistent, long-term NDVI data for the region were drawn from [27,31]. Average NDVI was calculated for 202 FMRs using Google Earth Engine based on five seasons in India—winter (December–February), spring (March–April), summer (May–June), monsoon (July–August), autumn (September–November)—and 15% cloud cover.

We applied quality mosaic in Google Earth Engine to reduce the set of NDVI layers during a specific season to a composite and to replace pixels represented as clouds with an accurately estimated pixel value by choosing the greenest pixel in the composite of multiple NDVI layers. The mosaicking method is especially useful for filling data gaps in Landsat 7 ETM+ images[32]. NDVI is commonly used by ecologists as a proxy for vegetation productivity especially in contexts where empirical vegetation data collection is limited due to high costs, time restrictions, and short climatic windows [27,31,33–35]. The index has shown consistent correlation with vegetation growth and biomass in various ecosystems worldwide [36,37]. NDVI is the ratio of the difference between the near-infrared (NIR) and red (R) bands and their sum [38]:

NDVI=((NIR−Red)/(NIR+Red)), [1]

where NIR is reflectance in the near-infrared band and Red is reflectance in the visible red band.

The NDVI algorithm takes advantage of the fact that green vegetation reflects less visible light and more NIR, whereas sparse or less green vegetation reflects a greater portion of the visible and less NIR. NDVI combines these reflectance characteristics in a ratio so it is an index related to photosynthetic capacity. The range of values obtained is between -1 and +1. Only positive values correspond to vegetated zones; the higher the index, the greater the chlorophyll content of the target species.

### Explanatory variables

Relevant social and ecological explanatory variables that relate to long-term vegetation growth trajectories were selected based on previous studies using a SES framework [13,28] with their measurable indicators constructed from available secondary social and spatial datasets (Tables A, B and C in S1 File). The choice of these variables reflects previous knowledge of the relative importance of such drivers in influencing the ecological outcomes as suggested by common property scholars studying Himachal Pradesh in the past [13,28,39].

We identified nine variables linked to the long-term vegetation outcome trajectories of the studied FMRs (Tables A, B and C in S1 File) and nested them in four SES dimensions (Actors, Governance System, Resource Unit, Resource System). We identified 24 indicators for the nine variables and quantified them based on local secondary social and spatial data. In addition, we identified three more variables based on secondary data: (*i*) Interactions (I), the reciprocal interactions between social and ecological subsystems based on forest fires; (*ii*) Related Ecosystems (ECO), attributes of related ecosystems, especially climatic factors of temperature, precipitation, and land surface temperature; and (*iii*) Outcome (O), the ecological performance of the SES in terms of average NDVI values.

### Constructing counterfactual trajectories

Synthetic control matching uses a nested optimization process and identifies a set of predictive weights for potential SES factors such that matching those weighted factors results in the closest possible match to NDVI outcomes over the full study period [23,25]. A plausible synthetic counterfactual provides the ability to assess the factors that structurally relate to the observed long-term trends in vegetation growth by re-creating those trends in the study FMR. To determine whether a particular synthetic counterfactual trajectory is plausible, it must closely match observed trends with minimum mean square prediction error, LOSS V (predictor weight matrix), and should be parallel [23–25]. We used nested optimization procedure developed for SCM and conducted our analysis in the Synth R package [40].

### Nested optimization

We constructed synthetic controls for the randomly selected set of 30 FMRs to get a set of critical factors that explain the observed long-term (2002–2016) outcome trajectories in our study area. Each of the 30 FMRs was assigned to a hypothetical policy intervention in 2017 (treated group), and the remaining 172 FMRs constituted a control (untreated) group for each treated FMR. Using nested optimization for SCM, explained below, we constructed counterfactual trajectories using a weighted combination of synthetically matched control FMRs. The analytical approach and the standard notation for the method is adapted from Abadie and Gardeazabal [25] and Abadie et al. [41], and implemented in the Synth R package[40]. The Synth package provides a data-driven procedure to create synthetic control units based on a weighted combination of control units that approximates the characteristics of the treated unit to allow for effect estimation of a policy intervention (treatment).

Let ‘*A*’ be a randomly drawn FMR from the total 202 FMRs in Kangra District. Let *K* be the total number of control FMRs (*K-A*) available for matching. In this approach, we compare the vegetation growth trajectory of ‘*A*’ with that of the weighted combination of control FMRs, chosen in such a way to resemble the characteristics of ‘*A*’. Such a weighted combination is used as a ‘*synthetic A*’.

Let W represent the weight assigned to each control FMR: W = (w_1_,….w_k_)’, a (*k x 1*) vector of nonnegative weights which sum to one. The reason for restricting W to nonnegative weights that sum to one is to restrict extrapolation within the support (convex hull) of the vegetation growth predictors for the control FMRs. In the synthetic control, we use a scalar *w*_*k*_ (*k* = 1,…, *K*) to indicate the weights assigned to control FMR *k* in ‘*synthetic A*’. W is chosen is such a way that ‘*synthetic A*’ closely matches the outcome trajectory of ‘*A*’.

Let *B*_*1*_ be a (*N x 1*) vector of *N* vegetation growth (NDVI) predictors/factors for FMR ‘*A*’. And, let *B*_*0*_ be an (*N x K*) matrix representing the values of the vegetation growth predictors for *K* possible control FMRs. Let V be a diagonal matrix with nonnegative values indicating the relative importance of the different vegetation growth predictors/factors in explaining the long-term outcome trajectories. The vector of weights W* is chosen in such a manner as to minimize

$$(B_{1}-B_{0}\;W)\prime V)\;(B_{1}-B_{0}\;W):w\;\epsilon \;Ʊ,$$ [2]

where

$$Ʊ=\{(w_{1},\ldots w_{k})’\;subject\;to\;w_{1}+..+..w_{k}=1\;and\;w_{k}\geq 0\;(k=1,2,\ldots K)\}.$$

W* is a function of V. The value of V could also be subjective and rely on prior knowledge of the relative importance of each vegetation growth predictor to the observed vegetation growth trajectory. Importantly, W* represents the combination of the control FMRs that best resemble ‘*A*’ FMR. SCM also yields a table comparing the vegetation growth predictors for ‘*A*’ and its ‘*synthetic A*’: B_1_* = B_0_ W*.

In Eq [3], we can estimate relative importance of the predictors/factors by calculating a solution to W*(V), which relies on diagonal matrix V whose diagonal elements give us weights reflecting the relative predictive importance of factors in B_1_ and B_0_. We follow the method of selection adopted by Abadie and Gardeazabal [25], who choose V in such a way that vegetation growth trajectory for ‘*A*’ is best reproduced by the resulting ‘*synthetic A*’.

For our study, let Y_1_ be a (*14 x 1*) vector containing the mean annual NDVI values for the treated FMRs during the 14-y period. Let Y_0_ be (*14 x K*) matrix with the same values of NDVI for the *K* potential control FMRs. Then

$$V^{*}=arg\;min\;{\left(Y_{1}-Y_{0}\;W^{*}(V)\right)}^{\prime }(Y_{1}–{Y_{0}}^{*}W\;(V))\;and\;V\;\epsilon \;ʋ,$$ [3]

where ʋ is the set of all nonnegative (diagonal) definite matrices of weights for the synthetic control. The weights for the synthetic control are depicted by W* = W* (V*), and the function synth () solves a nested optimization problem to minimize Eq 3, for W* (V) given by Eq 2, and to suggest a convex combination of control FMRs that has the lowest mean square prediction error[24,42]. Abadie and Gardeazabal [25]suggest normalizing the Euclidean norm of V* (or any of its positive diagonal elements) to one to solve for V*, given many possibilities for its solution. V* is a solution, so there are other solutions possible such as V*(s) = s. (V*) for any positive scaler value is s. Hence, normalizing the Euclidean norm of V* to one will provide a solution to Eq 3 and provide us with the weights reflecting the predictive importance of the chosen set of factors in explaining the long-term vegetation outcome trajectories.

The construction of the counterfactual trajectories using weighted combinations of synthetically matched control FMRs provides information on the different factors associated with the observed long-term vegetation trajectories. Out of 30 randomly selected and treated FMRs, we could obtain closely resembled vegetation growth trajectories between a random FMR and its synthetic control in 28 (Figures B-AE and Table E in S1 File). The list of selected counterfactual outcome trajectories and their mean square prediction errors and loss W (weights across control units) are given in Table E in S1 File. For each of the treated FMRs, we get V diagonal matrix (predictive weights) based on matching the random FMR and its synthetic control (Table F in S1 File). This matrix of V predictor weights permits the assessment of the relative importance of different factors in explaining the observed long-term vegetation growth trajectories for each FMR. Finally, we averaged the values of V for the 28 FMRs to list and rank predictors/factors that reproduced and explained the obtained vegetation growth trajectories (Table 1). To examine whether and how identified factors differed according to change in NDVI, we repeated this analysis and ranking for three levels (low, medium and high) of NDVI increase in the study FMRs (Table G in S1 File). We also checked the Synth R package–derived covariate balance for treated and control groups to select our final set of matched structural counterfactuals [40]. Higher covariate balance suggests well-matched treated and control groups, which further validates our approach.

**Table 1:** Predictive synthetic control matching (SCM) weights for social–ecological system (SES) indicators associated with long-term vegetation growth in Kangra District, Himachal Pradesh, India.

SES subsystem	Indicator	SCM weights (average)
***Actors***		
1. Users	Number of households	**0.09**
	Number of villages	0.02
	Number of farmers	0.03
	Number of marginal people	0.03
2. Socioeconomic conditions	Number of literates	**0.08**
	Number of unemployed people	0.03
	Economic activity	0.03
	Road density	0.03
3. Importance of resource	Number of small landholdings	0.03
***Governance System (planting program)***		
4. State afforestation programs	Forest area planted	0.02
	Broadleaf species planted	0.03
	Number of nurseries	0.03
***Resource Units and Resource System***		
5. Mobile animals	Number of grazing animals	0.02
6. Size of resource system	Forest beat area	0.02
	Tree cover	0.02
	Crop acreage	0.03
	Grass acreage	0.03
	Bare land acreage	0.03
7. System productivity	Soil depth	0.03
	Total carbon	**0.05**
	Total organic carbon	**0.04**
	Available soil water capacity	0.03
	Baseline vegetation (NDVI)	**0.12**
8. Location	Altitude	0.03
***Interactions***		
9. Conflicts among users	Number of forest fires	0.02
***Related Ecosystems (climatic factors)***		
10. Climatic factors	Temperature	**0.04**
	Precipitation	0.03
	Land surface temperature	0.03
Total predictive weights		1.00

Note: n = 28; values of key predictors in bold; see Table F in S1 File for synthetic weights for SES indicators for each of the 28 study FMRs.

FMRs are aggregate entities comprised of several villages. In such situations, a combination of comparison units (or synthetic control; a weighted average of all potential comparison FMRs) is usually a better option for reproducing the characteristics of units under study than any single comparison unit[23]. One important advantage of the SCM approach in tracing long-term outcome trajectories is that we expect FMRs that are similar in both observed and unobserved determinants of long-term vegetation growth, as well as their effect on this growth, should produce similar outcome trajectories over longer terms. Moreover, well-matched outcome trajectories of the unit of interest and their comparison units over of longer period helps control for unobserved factors as well as for the heterogeneity of the effects of the observed and unobserved factors on the outcome (vegetation growth) under study[23].

## Results

### Operationalizing a SES framework

This study used an SES framework to identify reciprocal interactions between forest users (Actors) and the planted or regenerated trees (Resource Units) inside the FMRs, and the institutional (Governance System) and ecological (Resource System) settings within which users and trees are embedded (Figure A in S1 File). Establishment of planted or regenerated trees (and resultant ecological outcomes) depends on interactions among forest users and managers within the larger social, economic, and ecological context. For example, specific Forest Department regulations affect these interactions, including rules promoting broadleaf species over conifers, an emphasis on afforestation of degraded landscapes to provide fodder, fuelwood, and other minor forest products, and expanding plantations via tree nurseries. However, successful tree establishment or regeneration in an FMR also depends on the socioeconomic and demographic attributes of resource users and the importance of the resource to the users.

Other key factors affecting long-term vegetation growth include the number of mobile (grazing) animals, size of the resource system, system productivity, geographic location, and climatic variables. In this Himalayan context, the occurrence of forest fires comprises a crucial reciprocal interaction between forest users and planted trees that influences long-term vegetation cover in the region[30,43]. Forest users usually burn forests to obtain better grass for their livestock, but burnt areas might influence ecological outcomes due to the direct loss of plants, which can influence long-term ecological outcome trajectories by promoting grass over trees (Figure A in S1 File). At the same time, the Forest Department seeks to control fires to support the vegetation improvement efforts it promotes. Successful management of this tree–grass tradeoff is critical for long-term vegetation growth in the region.

### Developing structural counterfactuals

**Fig 3:**
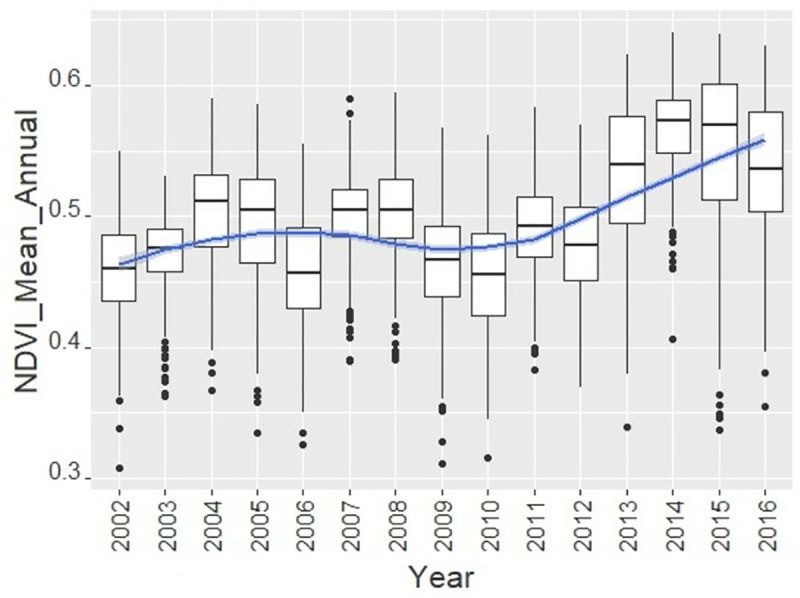
Vegetation growth trajectory (NDVI, mean annual) in 202 FMRs of the Kangra District.

Forest cover (Table D in S1 File) and vegetation more generally (Fig 3) exhibited an overall upward trend over the past two decades in Kangra District and the broader Himalayan region of India (27).

**Fig 4:**
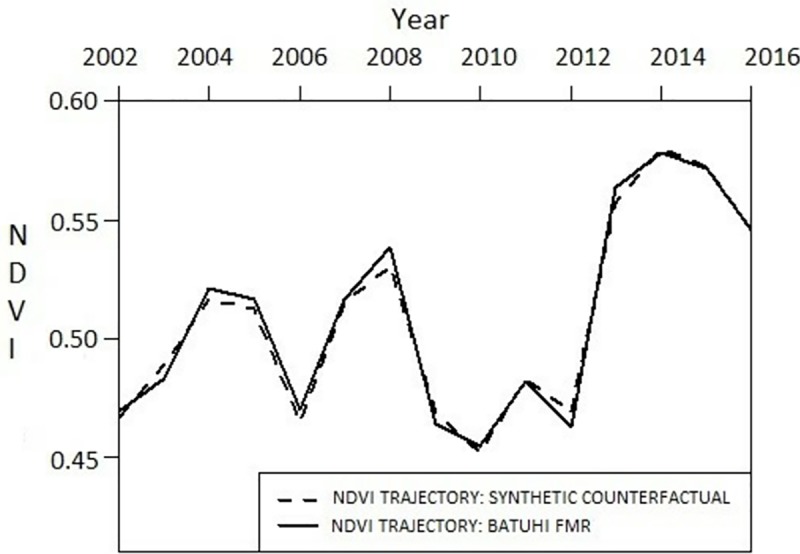
Illustrative matched synthetic counterfactuals for observed vegetation growth trajectory for Batuhi forest management unit (FMR), 2002–2016. Results for the remaining 29 randomly selected synthetically matched outcome trajectories provided in Figures B-AE and Tables E and F in S1 File.

Construction of a synthetic counterfactual trajectory matched to the observed vegetation (NDVI) trajectory suggests similarity between the factors in the two trajectories. Strong matches provide high confidence in the factors identified as determining long-term vegetation growth trajectories. The theoretically-informed predictors used in our analysis successfully reproduced the observed vegetation growth trajectories in nearly all the studied FMRs (28 of 30; 93.3%) over the period 2002 to 2016 as evidenced by minimum mean square prediction error, loss V (predictor weight matrix), and visual interpretation of trend, gap, and inflection points([24];Fig 4; Figures B-AE and Table E in S1 File).

### Predictors explaining ecological outcome trajectories

The average V predictor weights for the 28 FMRs with plausible synthetic outcome trajectories are listed individually in Table 1.

In the Actors subsystem of the SES framework, the number of households and number of literates were the two indicators with the highest predictive weights (0.09 and 0.08, respectively) in explaining observed long-term vegetation growth trajectories. The proportion of broadleaf species planted together with number of nurseries were the two Governance System (GS) indicators with the highest predictive weight. These two indicators relate to the strength of the Forest Department, a central governance actor in the region. The Department has long supported the implementation of afforestation programs, which is likely to influence long-term vegetation growth in interaction with local community participation in forest management (see Discussion below).

Predictive weights ranged from 0.02–0.12 under the Resource Unit and Resource System subsystems. Baseline vegetation, total carbon, and total organic carbon had the largest predictive weights among these subsystems in explaining observed vegetation growth. The occurrence of forest fires in an FMR had a predictive weight of 0.02. Finally, climatic factors (temperature, precipitation, land surface temperature) also had higher relative importance in explaining observed vegetation trajectories with predictive weights of 0.03–0.04. Overall, baseline vegetation, the number of households, number of literate people, temperature and soil quality (carbon and organic carbon) had the largest predictive weights (∃ 0.04) in explaining the observed outcome trajectories.

We also found spatial heterogeneity in vegetation growth with some FMRs experiencing much higher level of NDVI increase than other FMRs (Table G in S1 File). Several predictors increased in importance as levels of change in NDVI growth increased. These include: number of households, number of marginal people, number of literates, forest area planted, soil quality (carbon and organic carbon), temperature and baseline vegetation. By contrast, nearly all of the indicators with greater predictive importance (i.e. those in bold in Table G in S1 File) in low-growth FMRs (<10%) decreased in importance in the higher growth trajectory FMRs (>10%). Only one FMR had less than 10% increase in NDVI (Ghoran). In this case, number of unemployed people, crop acreage, economic activity and number of farmers emerged as more important predictors in shaping the long-term vegetation growth (Table G in S1 File).

## Discussion

### Socioeconomic factors

The number of households and level of literacy in FMRs were the social factors with the highest ability to predict the observed NDVI trajectories (∃ 0.08; Table 1). This finding held in analyses of those FMRs that experienced higher vegetation growth levels (i.e. >10%; Table G in S1 File). Population and livelihood changes in the region over the past two decades suggest a shift toward lower dependence on agriculture and forests, which may help explain increasing vegetation growth. Dependence on agriculture as the main local livelihood activity declined considerably during 2005–2011 (Table H in S1 File) while number of households, number of unemployed people, and level of literacy increased. For our matched 28 FMRs, we found a reduction of 176 farming households per FMR during the study period, which indicates a decline in agriculture in the area. Moreover, 19 of the matched FMRs saw an increase in literacy, on average, which is likely associated with a shift towards non-farm based occupations in the region [13,28] as more generally [44] (Table I in S1 File). These changes support our expectation that socio-economic development (e.g. through greater literacy levels and off-farm job opportunities) has triggered agricultural abandonment thereby increasing vegetation growth in Kangra District [13,28,45].

The “number of households” variable represents resource users who live in a FMR and use its forest resources for subsistence and commerce. In the study context, the use of forest resources is mainly for fuelwood, fodder, and grazing needed to support a subsistence, livestock-based economy. The increase in number of households largely reflects the division of family farms due to transfer from parents to heirs. The decline in farm size and low labor availability pose challenges for agriculture in many parts of Kangra District, which may led to reductions in cultivated area and concomitant increases in vegetation. Our findings contrast to other research in the region [28], which has found a negative association between the number of households and forest condition. However, this earlier work was based on cross-sectional data whereas our study covers a 15-y time period, which provides a more expansive portrait of the relationship between the number of households and vegetation dynamics.

The predictive importance of factors in our SES framework varied according to level of vegetation growth. A different set of factors had more predictive power in low vegetation growth FMRs (< 10% increase in NDVI from the baseline year) compared to medium and higher growth FMRs (e.g. >10%). Broader trends in the region (Table H in S1 File) suggest potential explanations for this variation, but further research is needed to understand why certain factors (socio-economic ones as well as governance and biophysical ones) have more predictive capacity than others at different points on the forest transition curve[46].

### Forest governance factors

Governance factors were also important in predicting positive vegetation outcome trajectories in the region. Together, our three governance indicators—forest area planted, percentage of broadleaf species planted, and number of nurseries—predicted 8% of the vegetation change seen over the study period (Table 1). By contrast, governance indicators were much less important in low vegetation growth FMRs, with area planted and number of nurseries having no predictive relationship to NDVI outcomes.

Widespread community opposition and protests led the government of India to adopt a Joint Forest Management policy in the 1990s, which promoted forest plantations to better address community needs compared to previous state-led pine monocultures. This policy emphasized expanding plantation area, increasing the number of nurseries, and including a greater proportion broadleaf species. An emphasis on more economically-valuable broadleaf species such as *Acacia catechu* and *Dalbergia sissoo* in forest plantations has encouraged more community involvement in tree planting and management. In turn, this involvement led to local support that has ultimately spurred positive vegetation growth trajectories in the region [28,47].

### Biophysical factors

Soil quality parameters also had relatively high predictive values (0.04–0.05) in explaining the observed NDVI outcome trajectories (Table 1). Presence of higher total soil carbon and organic carbon indicates the productive potential of the soils, which can trigger increased vegetation growth over the long term provided such land is not diverted to agricultural expansion. Organic carbon provides structural stability to soils, essential for optimum growth of plants: stable pores and particles store water, transmit water and air, and provide space for roots to grow [48].

Baseline vegetation status was the strongest determinant of long-term positive NDVI outcome trajectories overall, although results varied by the level of NDVI increase (Table G in S1 File). For FMRs with an increase in NDVI ≤ 10%, baseline vegetation had moderate predictive importance, but for FMRs with an increase > 10%, baseline vegetation was among the top predictive factors. These results suggest the importance of initial forest conditions in shaping long-term outcomes. Baseline vegetation has less predictive importance in FMRs with lower NDVI increases as social and economic factors (e.g. number of unemployed people, crop acreage, economic activity, number of farmers) played a more important role in triggering vegetation growth—likely in part precisely because there was a lower baseline vegetation status to begin with so more concerted effort would be required to increase it (Table G in S1 File). This finding may reflect intensive use of a given FMR’s resources due to higher reliance on forest resources. For example, higher local unemployment and a larger number of farmers may lead people in a given FMR to rely on agricultural-based livelihoods that imply, in this context, use of forest resources. In turn, use of land for agriculture or consumption of forest resources may lead to lower increases in NDVI.

High baseline vegetation implies more tree cover, which invites monitoring and enforcement from local forest officials thereby resulting in better long-term vegetation growth. In our matched sample, one FMR (Mangwal) recorded NDVI decrease despite having good baseline vegetation status (greater than 0.456 NDVI, average, n = 28 matched) (Table I in S1 File). Factors such as forest fires and infrastructure development are likely to explain the relatively low NDVI increase in this FMR. On the other hand, two FMRs having poor baseline vegetation status either recorded decrease (Hagwal) or low NDVI increase (Ghoran) (Table I in S1 File). Factors such as higher forest dependence due to absence of off-farm opportunities, infrastructure development and ineffective governance are the likely reasons that explain poor NDVI growth outcomes in these cases[13,28].

For FMRs with NDVI increases up to 10%, crop and grass acreage, bare land acreage, altitude, and land surface temperature were the factors with high predictive importance in reproducing the observed outcome trajectories (Table G in S1 File). For FMRs with an NDVI growth increase > 10%, grass acreage and temperature were the main predictors with high relative importance (∃ 0.04). This finding may be due to fragmentation in land uses over time wherein planted forest area increases while livestock-based livelihoods increasingly rely on separate pasture land rather than clearing more forest land or using forest resources for fodder. This transition coupled with trends toward abandonment of agricultural fields may lead to co-occurrence of greater area in pasture and in forest. Another reason higher grass acreage may be associated with more vegetation growth is because open grasslands represent an obvious target for state-led afforestation programs and communities may be motivated to avoid state incursion by maintaining some level of tree cover on communal lands.

More cropped areas usually translate to more resource users, which may indicate higher dependence on forests for subsistence needs. On the other hand, more bare land acreage may suggest more opportunities for extending tree cover. However, it may also indicate presence of unproductive land, which may be unfit for vegetation growth due to poor soil and rocky landscapes. Higher elevations offer limited potential for vegetation growth due to cold temperatures and lack of deep soils. Warmer temperatures may lead to increase in vegetation growth due to positive effects on the rate of plant growth and expansion into other areas. Temperature may affect plant growth depending on the location, site quality, and exposure range and duration [49,50].

### Potential limitations

Our use of publicly accessible secondary data means there were limitations in the indicators available for our analysis. Our results suggest we were able to identify suitable indicators for the different components of the SES framework we employed, but it is possible that our analysis omitted some important potential indicators. For example, we were not able to explore the potential predictive importance of village-level institutional and governance factors such as efficacy of monitoring and enforcement, community self-organization, and community institutional strength and tenure, all of which have been shown to be important in shaping common pool resource outcomes [10,11,51]. SES are also complex, involving feedbacks [52,53] and interactions across scales [54,55]. Our analytical approach appears to account for these complexities given its ability to accurately trace the long duration vegetation growth trajectory in the study context [23], but it may have missed some interactions and pathways leading to observed long-term ecological outcomes [11,56]. Finally, the nested optimization-derived factors in our analysis are predictive rather than causal in nature. Further research is required to evaluate the causal relationships between key factors and long-term outcomes.

## Conclusion

This study has shown that our approach, based on the synthetic control method in an SES framework, can help identify a set of predicting factors that have higher relative importance in explaining observed vegetation trajectories in SESs. We found a suite of socioeconomic, forest governance and biophysical factors that have shaped long-term ecological outcomes in Kangra district of Himachal Pradesh. Our results also showed considerable heterogeneity in vegetation outcomes and predictors based on level of NDVI increase in FMRs.

This study has at least three important implications for future research and policy. First, it demonstrates the potential to use publicly available data to explore long-term social and ecological trends in a wide array of contexts, including those where data collection is otherwise difficult or costly. Second, our approach used with data from different contexts can help advance theory on effective governance of natural resources across a range of SESs. For example, comparative analyses can help identify key enabling conditions and factors that consistently influence long-term outcomes across different contexts. Our approach can also be used to build and test structural models capable of elucidating casual relationships in coupled human and natural systems. Here, we have focused on NDVI as the key ecological outcome, but our approach can be used to examine both social and ecological outcomes.

Finally, our approach can be used to help target new programs and policy interventions in SESs as well as to evaluate the long-term impacts of previous interventions. By identifying and ranking relevant variables in a given SES, analysts and programmers will have information about conditions that may be more or less propitious for new resource management programs and policies and can tailor such interventions accordingly. For previous or on-going interventions, synthetic control matching can be extended to assess impact, including over longer periods of time. Our approach can also be used to identify counterfactual trajectories that can serve as reference scenarios for evaluating the performance of environmental policies against their stated objectives.

## Supporting information

S1 File This file includes Figures A-AE, Tables A-I and references for supporting information citations.
(DOCX)
