# Supplementary material for: Explaining long-term outcome trajectories in social–ecological systems
Source: PLoS One. 2019 Apr 15;14(4):e0215230. doi: 10.1371/journal.pone.0215230 (PMC6464167; doi:10.1371/journal.pone.0215230)
Supplement: S1 File — This file includes Figures A-AE, Tables A-I and references for supporting information citations. (DOCX) [file pone.0215230.s001.docx]

**Supplementary Information** for

**Explaining long-term outcome trajectories in social–ecological systems**

**S1 File:** This file includes Figs. A-AE, Tables A-I and references for supporting information citations

**Fig. A.** Operationalizing a social–ecological system (SES) framework [1]to predict vegetation growth trajectories with forest fires as the reciprocal interaction. Planted enclosures (with seedlings, RU) or regenerated areas are part of FMRs and provide locations for forest fires (SES interaction), which are often initiated by local people to promote new grass growth. FMRs set conditions or constraints for the severity and extent of forest fires and the feedbacks among FMRs, forest fires, and planted/regenerated areas. These interactions ultimately shape the vegetation growth trajectories in the FMRs over space and time. The Forest Department (Governance System in the SES framework) defines rules and policies relating to fires and resource use by forest users (Actors) within FMRs. Favorable long-term vegetation growth depends on successful management of dynamic interactions and feedbacks among forest users and planted/regenerated areas.

**Social, Economic and Political Settings (S)**

***Social-Ecological System***

Forest management regions (FMRs), **Resource System**

Forest department programs/policies

**Governance System**

*Feedback*  *Feedback*

***Set conditions for Set conditions for***

***Are part of Forest fires Vegetation growth* Define and set**

**Interaction (I) Outcomes (O) rules for**

***Are inputs to Participate in***

Forest users

**Actors**

Planted seedlings or regenerated area inside FMR

**Resource Units**

***Related Ecosystems (ECO)***

**Fig. B-Fig. AE.** Matched synthetic counterfactuals for observed vegetation growth trajectories (2002–2016) in 30 randomly selected forest management regions (FMRs) in Kangra District, Himachal Pradesh, India. Two FMRs, Jol and Kandi, are shaded to indicate that they were not selected for analysis due to their nonplausible counterfactuals.

**Fig. B. Balakh Fig. C. Bassa**

**
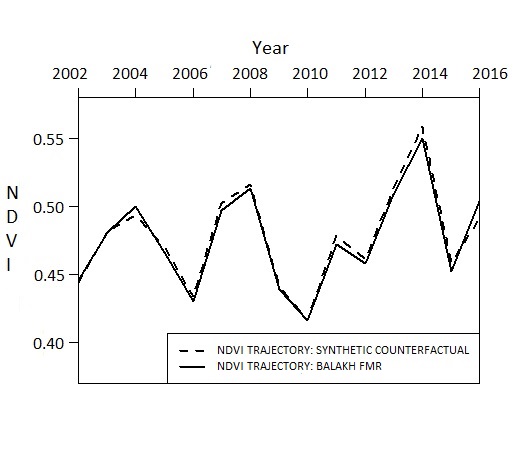

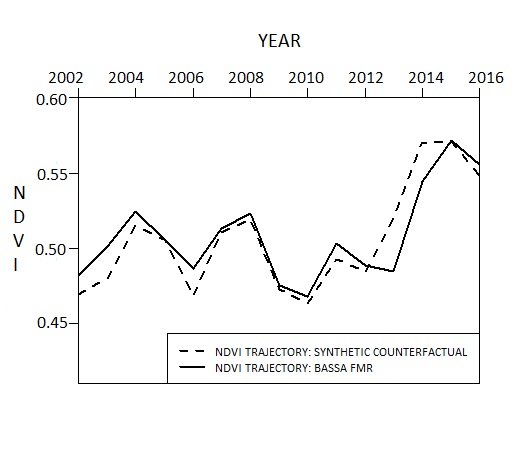
**

**Fig. D. Batuhi Fig. E. Bindrawan**

**
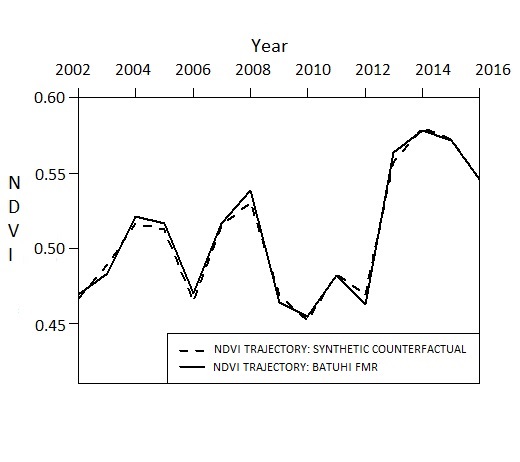

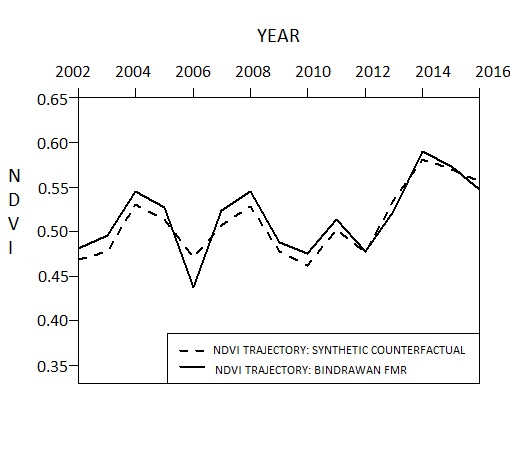
**

**Fig. F. Borka Fig. G. Daulatpur**

**
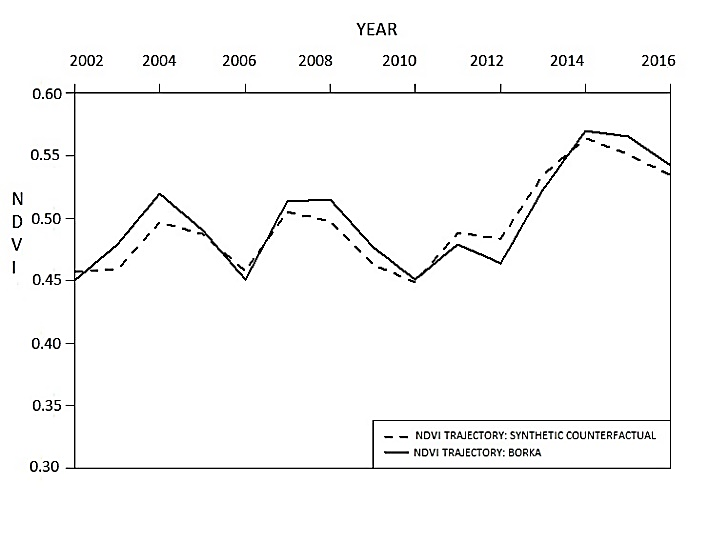

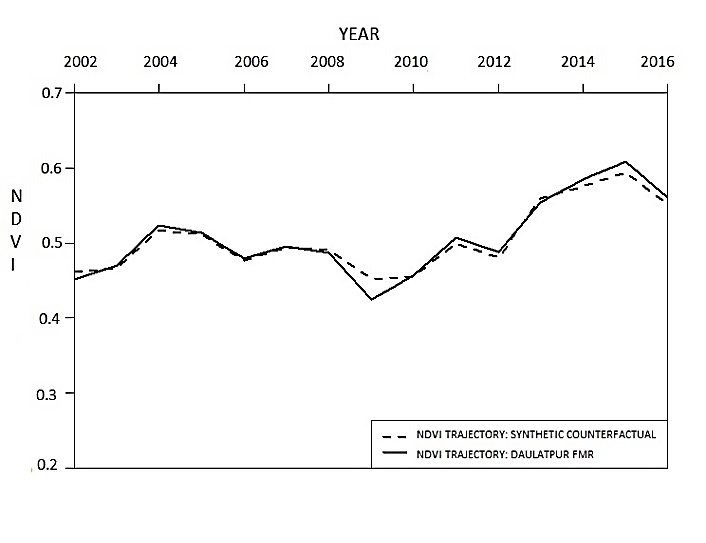
**

**Fig. H. Fatehpur Fig. I. Ghoran**

**
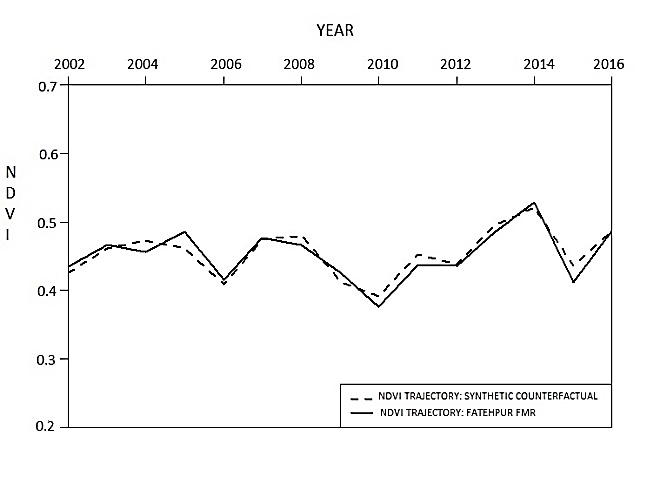

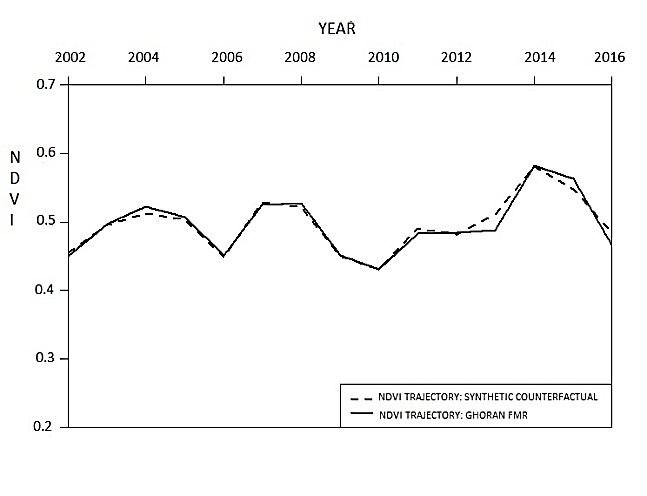
**

**Fig. J. Gurial Fig. K. Hagwal**

**
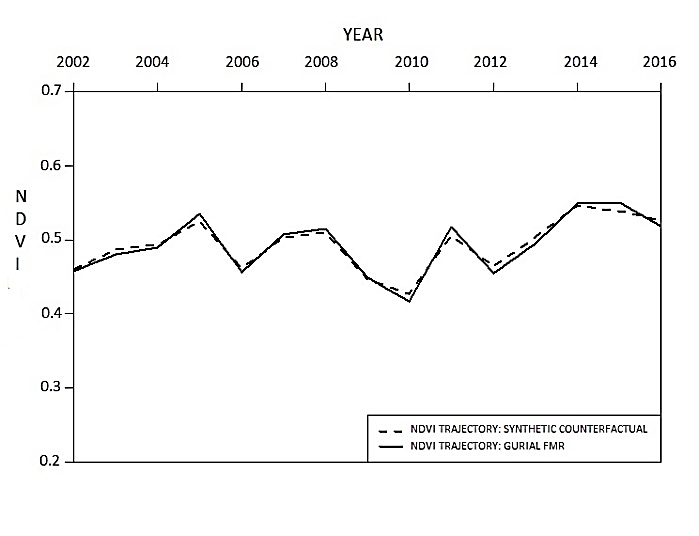

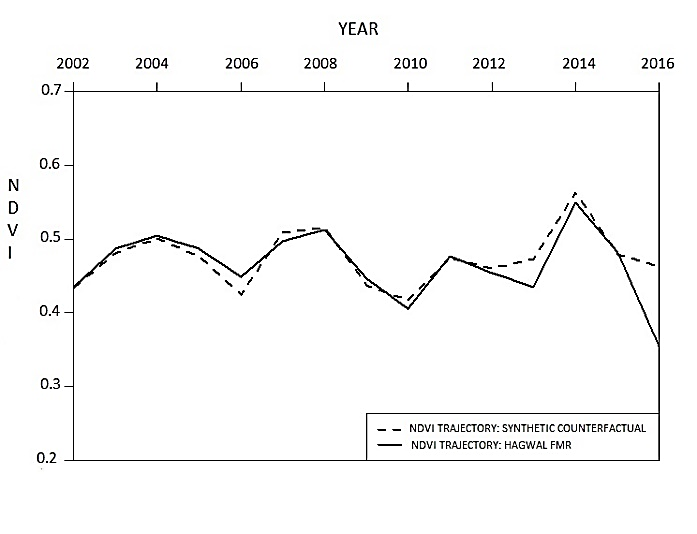
**

**Fig. L. Jaisinghpur Fig. M. Jalari**

**
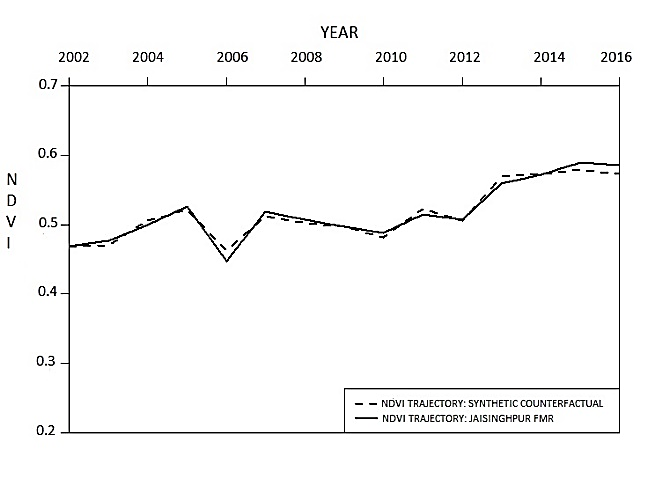

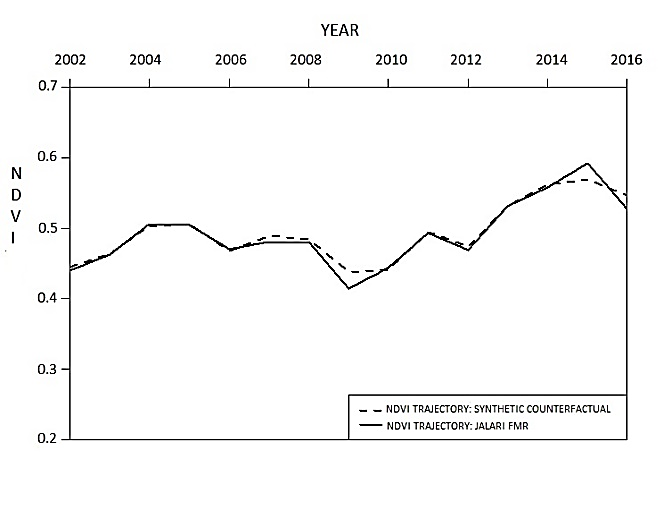
**

**Fig. N. Jol Fig. O. Kandi**

**
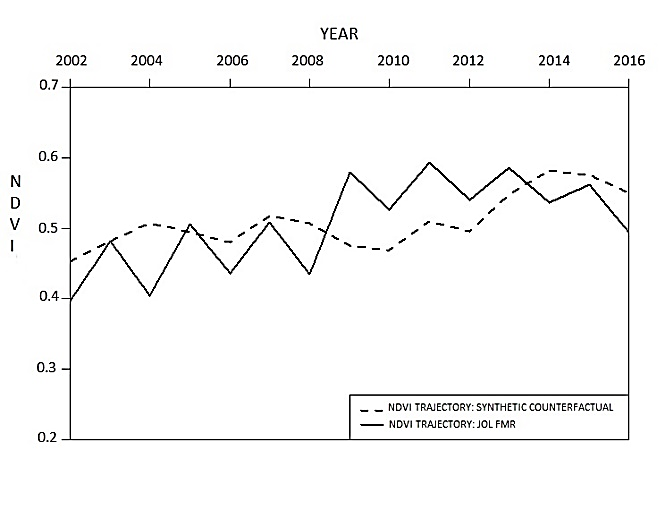

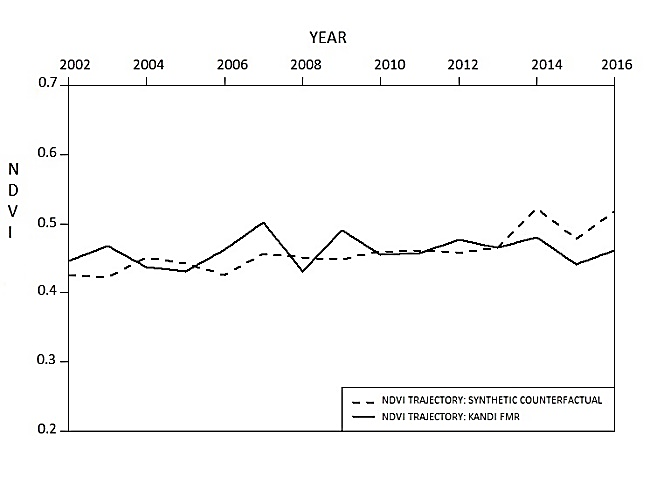
**

**Fig. P. Khabbal Fig. Q. Khanni**

**
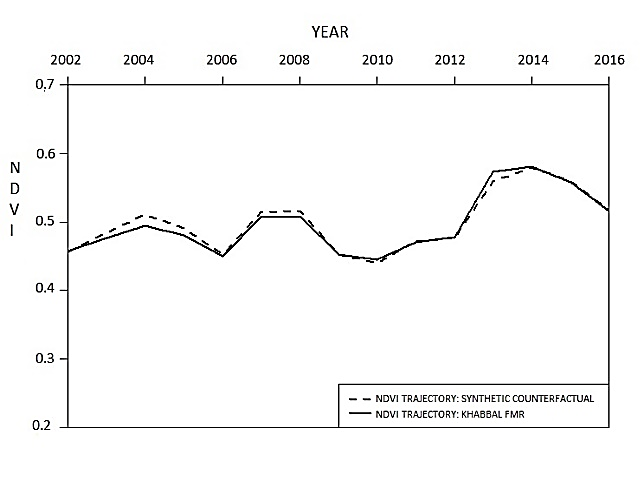

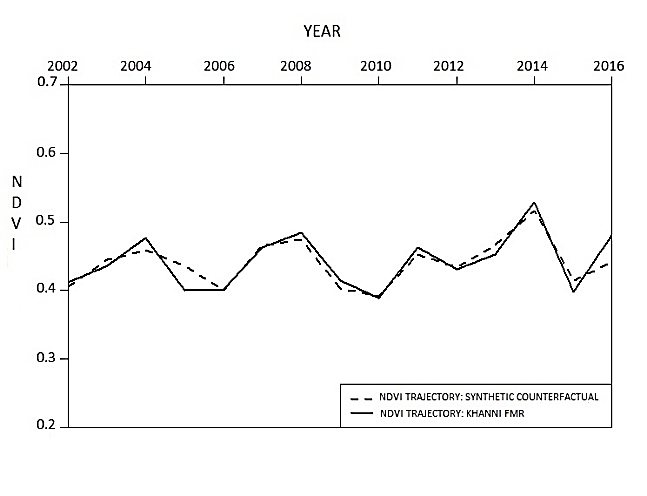
**

**Fig. R. Khanpat Fig. S. Kothi**

**
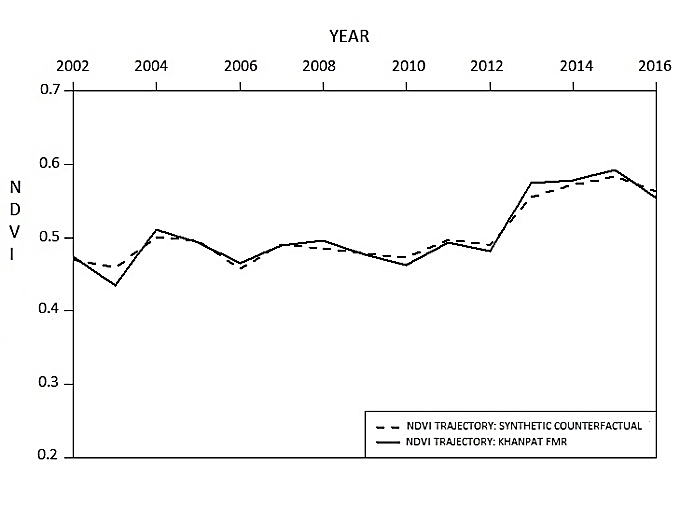

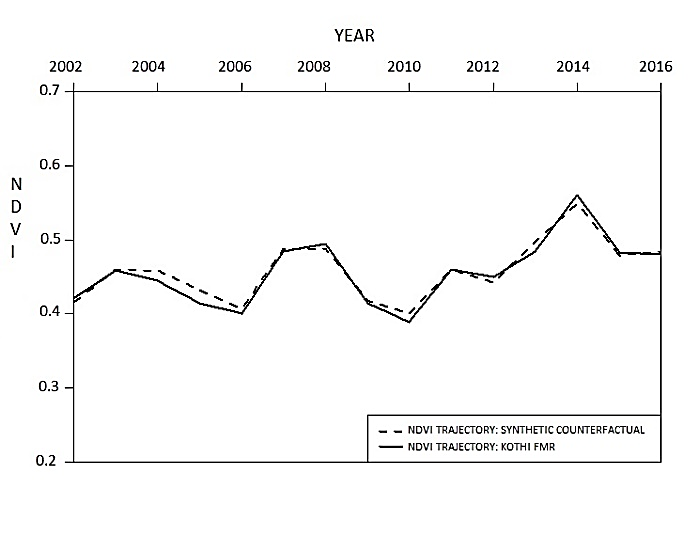
**

**Fig. T. Lahat Fig. U. Maira**

**
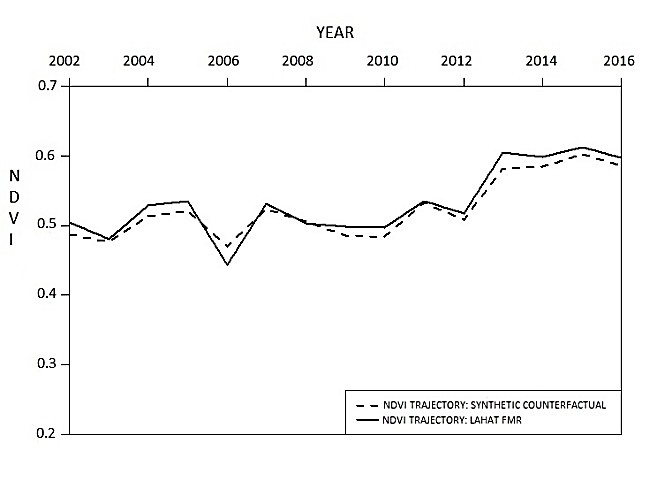

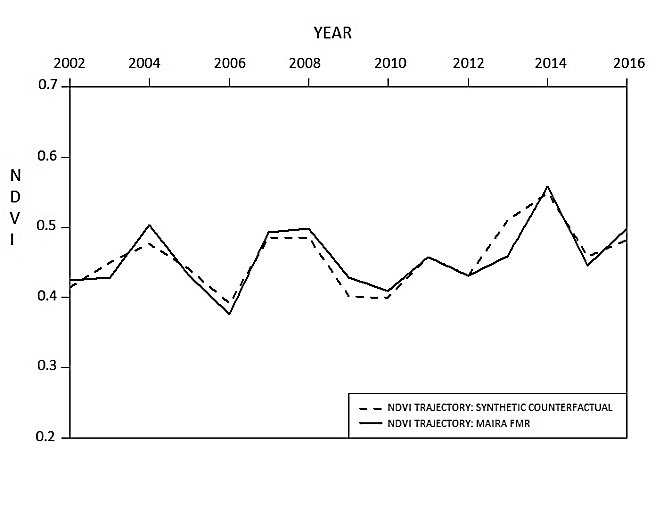
**

**Fig. V. Mangwal Fig. W. Nangal**

**
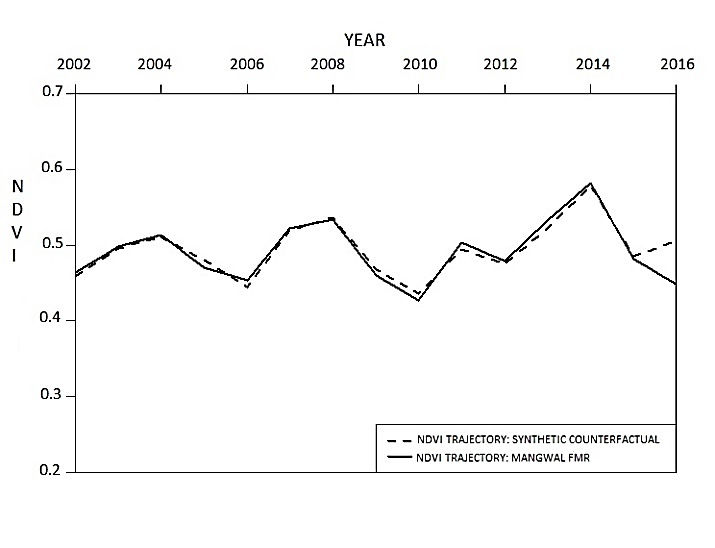

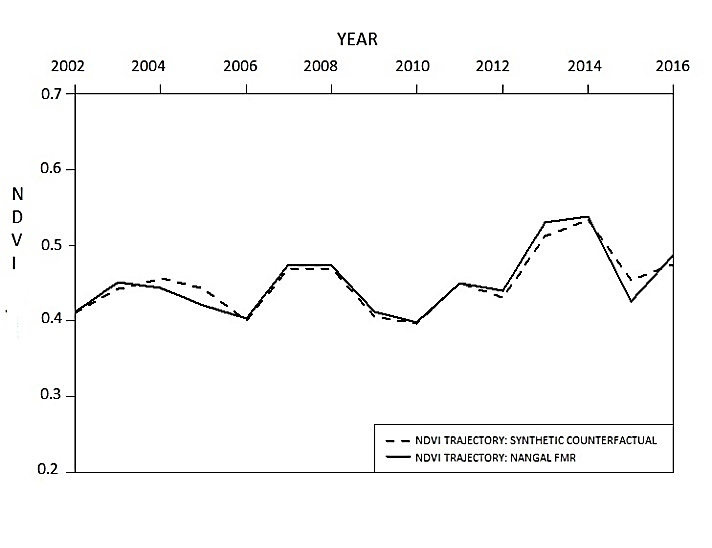
**

**Fig. X. Ramehar Fig. Y. Rehlu**

**
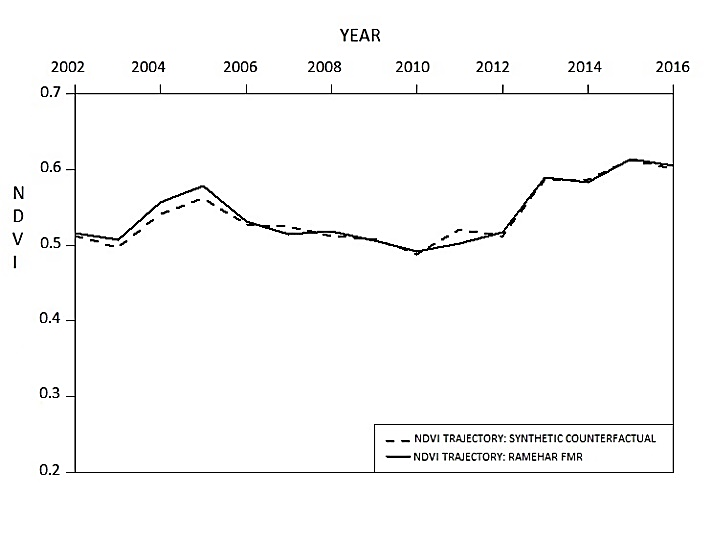

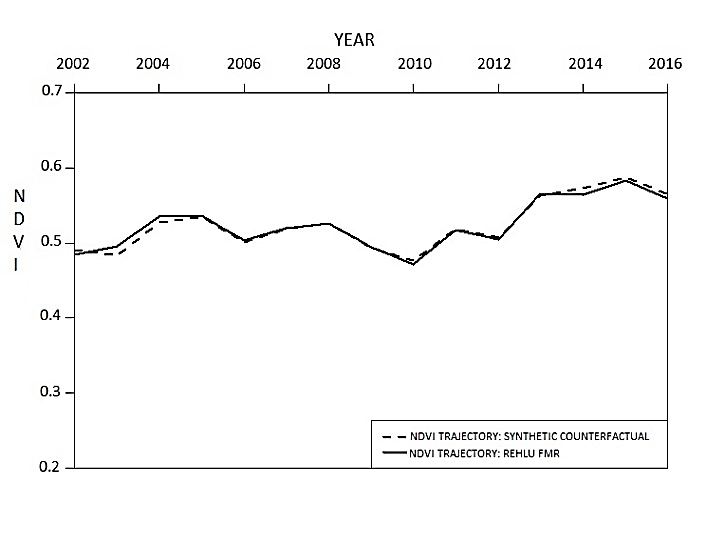
**

**Fig. Z. Rirkmar Fig. AA Salol**

**
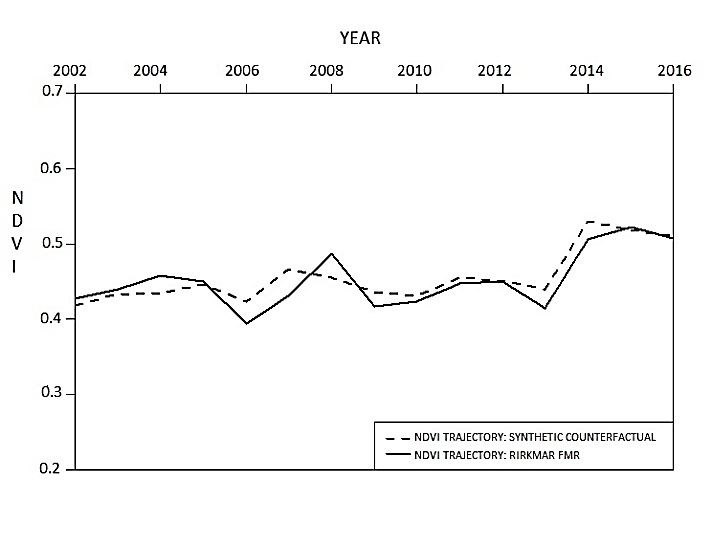

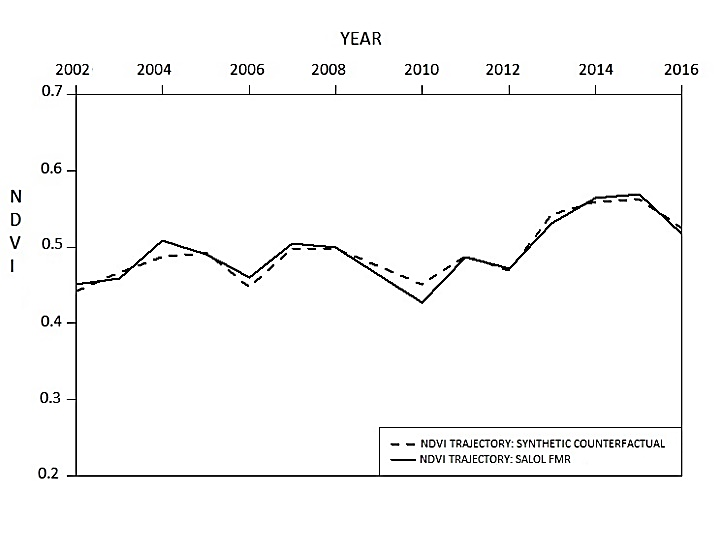
**

**Fig. AB Satobari Fig. AC. Soldha**

**
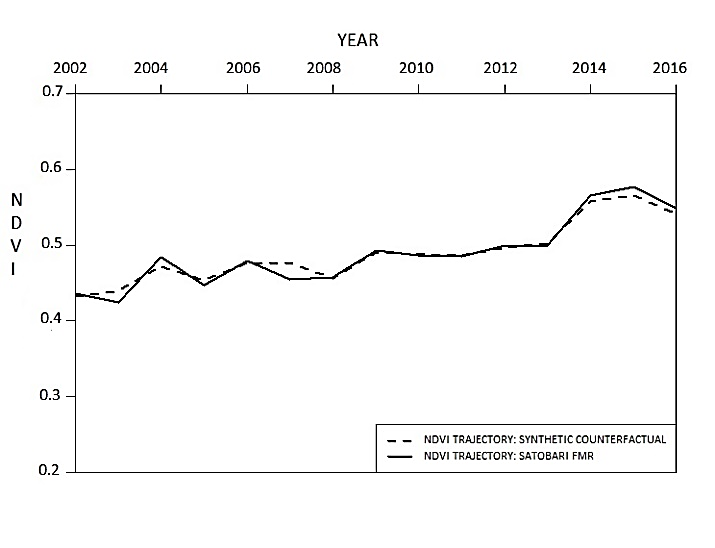

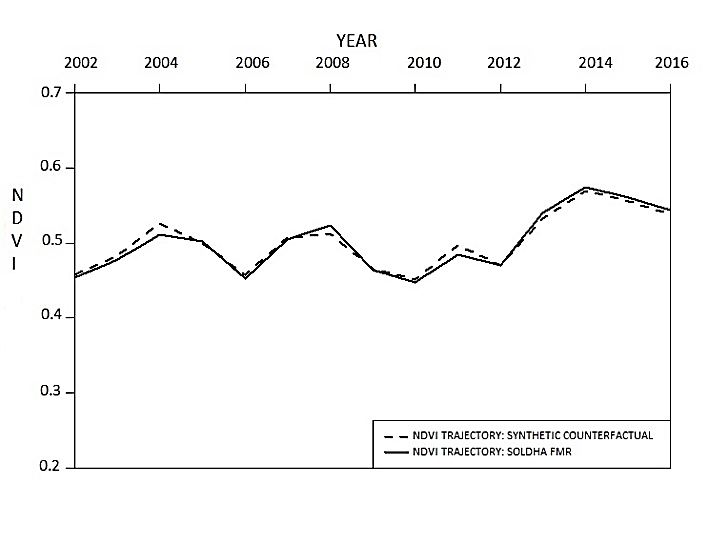
**

**Fig. AD. Sunhi Fig. AE. Talara**

**
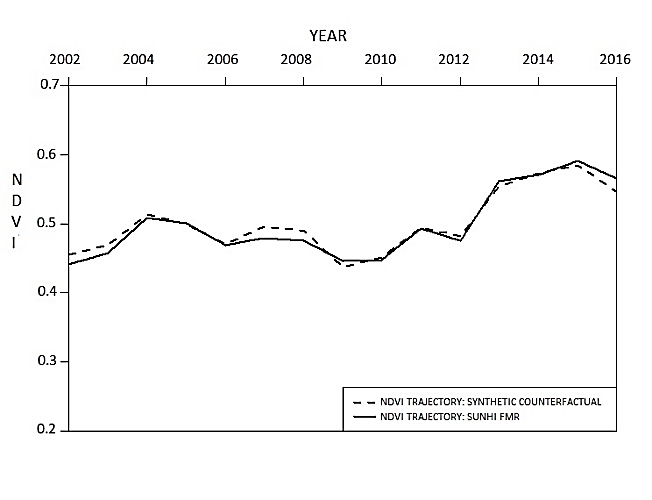

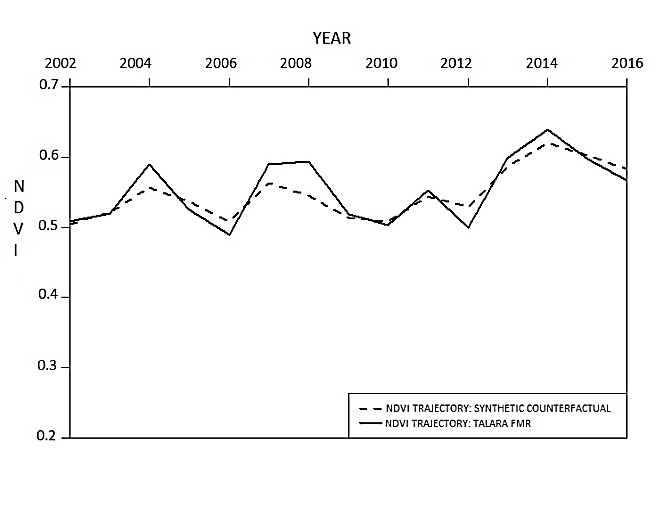
**

**Table. A** Social–ecological system study variables related to long-term ecological outcomes in forest management regions, Kangra District, Himachal Pradesh, India (n = 202)

| **Variable** | **Indicators** | **Mean** | **Std. Dev.** | **Range** |
| --- | --- | --- | --- | --- |
| **Subsystem 1: Actors (A)** |  |  |  |  |
| 1. Users | Number of households | 1080 | 1176 | 7220 |
|  | Number of villages | 13 | 10 | 59 |
|  | Number of farmers | 317 | 333 | 1919 |
|  | Number of marginal people | 1067 | 1228 | 9176 |
| 2. Socioeconomic conditions | Number of literate people | 3685 | 4046 | 23299 |
|  | Number of unemployed people | 1011 | 973 | 5645 |
|  | Economic activity (1-63, values) | 5.05 | 3.82 | 24.13 |
|  | Road density (km/km^2^) | 1.11 | 0.48 | 2.48 |
| 3. Importance of resource | Number of smallholdings | 921 | 871 | 5313 |
| **Subsystem 2: Governance System (GS)** | |  |  |  |
| 4. State afforestation programs | Area planted (ha) | 80.48 | 58.57 | 498 |
|  | Broadleaf sp. Planted (Percent) | 38.36 | 15.79 | 78.92 |
|  | Number of nurseries | 0.27 | 0.52 | 3 |
| **Subsystems 3 &4: Resource Units and Resource System (RU and RS)** | |  |  |  |
| 5. Mobile animals | Number of grazing animals | 4970 | 3805 | 21812 |
| 6. Size of resource system | Forest beat area (ha) | 1756.14 | 1031.04 | 5570.41 |
|  | Tree cover (ha) | 1037.7 | 531.13 | 3101.31 |
|  | Crop acreage (ha) | 41.44 | 22.97 | 94.74 |
|  | Grass acreage (ha) | 16.47 | 9.53 | 48.94 |
|  | Bare land acreage (ha) | 0.97 | 3.09 | 19.84 |
| 7. System productivity | Soil depth (cm) | 95.1 | 20.47 | 90 |
|  | Total carbon (Kg C m-2) | 7.58 | 1.62 | 5.97 |
|  | Total organic carbon (% weight) | 1.36 | 0.34 | 1.38 |
|  | Available soil water capacity (mm) | 99.58 | 36.78 | 135 |
|  | Baseline vegetation/NDVI (-1 to 1) | 0.5 | 0.04 | 0.21 |
| 8. Location | Altitude (m) | 874.79 | 562.53 | 2508.24 |
| **Interactions (I)** |  |  |  |  |
| 9. Conflicts among users | Number of forest fires | 1.96 | 3.11 | 25 |
| **Outcomes (O)** |  |  |  |  |
| 10. Ecological performance | NDVI (-1 to 1) | 0.5 | 0.06 | 0.33 |
| **Related Ecosystems (ECO)** |  |  |  |  |
| 1. Climatic factors | Temperature (Degree Celsius) | 18.26 | 3.75 | 14.69 |
|  | Precipitation (mm) | 77.09 | 2.86 | 16.38 |
|  | Land surface temperature (Kelvin) | 297.05 | 3.62 | 17.56 |

**Table. B.** Descriptive information on predictive social and ecological variables used to analyze outcome trajectories in forest management regions (FMRs) in Kangra District, Himachal Pradesh (HP), India (values are at FMR level).

| **Variable** | **Description** | **Unit of measurement (average at FMR level)** | **Sources of data** |
| --- | --- | --- | --- |
| ***Subsystem 1: Users*** |  |  |  |
| 1. Number of households | Number of households | Number | Census (2001, 2011), India, <http://censusindia.gov.in/> |
| 1. Number of villages | Number of villages | Number | Census (2001, 2011), India, <http://censusindia.gov.in/> |
| 1. Number of farmers | Number of farmers | Number | Census (2001, 2011), India, <http://censusindia.gov.in/> |
| 1. Marginal population | Scheduled caste population | Number | Census (2001, 2011), India, <http://censusindia.gov.in/> |
| 1. Number of literates | Total number of literates | Number | Census (2001, 2011), India, <http://censusindia.gov.in/> |
| 1. Unemployment | Total marginal workers | Number | Census (2001, 2011), India, <http://censusindia.gov.in/> |
| 1. Economic activity | 1992–2013, 0.56 km spatial resolution | 1 to 63 (values) | Version 4 DMSP-OLS Nighttime Lights Time Series |
| 1. Road density | Road density, 2013 | Pixel 30x30 meter and unit km/km^2^ | HP Forest Department (GIS cell data) |
| 1. Number of small landholdings | Number of small land-holdings less than 0.5 ha | Number | Agricultural census (2005, 2011), India |
| ***Subsystem 2: Governance*** | |  |  |
| 1. Government plantation program |  |  |  |
| - Plantation policy (area planted) | Total area planted in hectares | Hectares | Dharamsala Forest Circle Forest records, HP Forest Department, India |
| - Plantation policy (broadleaf sp. planted) | Percentage of Broadleaf species planted | Percent | Dharamsala Forest Circle Forest records, HP Forest Department, India |
| - Plantation policy (number of nurseries) | Number of nurseries | Number | Dharamsala Forest Circle Forest records, HP Forest Department, India |
| ***Subsystem 3: Resource Units*** | |  |  |
| 1. Mobile animals (number of grazing animals) | Number of grazing animals (buffaloes, goats, sheep, cattle) | Number | Livestock census (2007, 2012), India |
| ***Subsystem 4: Resource System*** | |  |  |
| 1. Size of resource system (area of forest beat) | Area of forest beat | Hectares | Dharamsala Forest Circle Forest records, HP Forest Department, India |
| 1. Size of resource system (tree cover) | Tree cover, 2000 | Hectares | Hansen et al. (2013) Global Forest Change Data, FAO (15% definition) |
| 1. Size of resource system (crop acreage) | 2001–2015, 30 m resolution | Hectares | Land cover types, GlobeLand30 (GL30), Chen et al. (2015)[2] |
| 1. Size of resource system (grass acreage) | 2001–2015, 30 m resolution | Hectares | Land cover types, GlobeLand30 (GL30), Chen et al. (2015)[2] |
| 1. Size of resource system (bare land acreage) | 2001–2015, 30 m resolution | Hectares | Land cover types, GlobeLand30 (GL30), Chen et al. (2015)[2] |
| 1. System productivity (soil depth) | 2000, reference soil depth  AVERAGE | cm | Regridded Harmonized World Soil Database v1.2; [3]  <https://daac.ornl.gov/SOILS/guides/HWSD.html> |
| 1. System productivity (total carbon) | 2000, topsoil + subsoil carbon content  AVERAGE | kg C m-2 | Regridded Harmonized World Soil Database v1.2; [3]  <https://daac.ornl.gov/SOILS/guides/HWSD.html> |
| 1. System productivity (total soil organic carbon) | 2000, topsoil +subsoil organic carbon  AVERAGE | % weight | Regridded Harmonized World Soil Database v1.2; [3]  <https://daac.ornl.gov/SOILS/guides/HWSD.html> |
| 1. Location (altitude) | 2000, 90 m resolution | Meter | SRTM (Shuttle Radar Topography Mission), 90 m resolution, 2000 |
| 1. System productivity (available soil water capacity) | 2000, available soil water storage capacity AVERAGE | Coded values 1 to 7; 1 = 150 mm water per m of the soil unit, 2 = 125 mm, 3 = 100 mm, 4 = 75 mm, 5 = 50 mm, 6 = 15 mm, 7 = 0 mm. | Regridded Harmonized World Soil Database v1.2; [3]  <https://daac.ornl.gov/SOILS/guides/HWSD.html> |
| 1. System productivity (baseline vegetation or NDVI) | 2002–2016, 30 m resolution | -1 to 1 (range), annual mean NDVI value for a FMR is an average for all five seasons in the study area. For example, for mean annual NDVI for y 2002 = Average (Winter NDVI (Dec 2001 to Feb 2002), Spring NDVI (March and April 2002), Summer NDVI (May and June 2002), Monsoon NDVI (July and August 2002) and Autumn NDVI (Sept to November 2002). | Landsat 7, 8, |
| ***Interactions (I) -> Outcomes (O)*** | |  |  |
| 1. Conflicts among users (forest fire occurrences) | 2002–2016 | Number | NASA, active fire data, MODIS C6 |
| ***Outcomes (O)*** |  |  |  |
| 1. Ecological performance measure (NDVI) | 2002–2016, 30 m resolution | -1 to 1 (range) | Landsat 7, 8 |
| ***Related ecosystems (ECO)*** | |  |  |
| 1. ECO1 |  |  |  |
| - Temperature | 2001–2015, 30 m resolution | Degree Celsius | CRU (Climatic Research Unit) TS dataset |
| - Precipitation | 2001–2015, 30 m resolution | Millimeter | CRU (Climatic Research Unit) TS dataset |
| - Land surface temperature | 2001–2015, 5.5 km spatial resolution | Kelvin | MODIS/Aqua Land Surface Temperature/Emissivity Monthly L3 Global CMG V005 |

**Table. C.** Theory of change linking each factor with long-term vegetation growth in forest management regions (FMRs) in KSES.

| **Variable** | **Hypothesized causal relationships of predictors to long-term vegetation outcome trajectories with indicative references.** |
| --- | --- |
| **First-level core subsystems: Social, economic, and political settings (S)** | |
|  |  |
| **Subsystem 1: Actors (A)** |  |
| 1. Number of households | Chhatre and Agrawal (2008) found a slight positive relationship between number of forest users and regeneration after controlling for other factors. People in the study area meet some portion of their energy needs with fuelwood collected from forests. We expect higher fuelwood dependence with increasing number of forest users [4].  Chhatre and Agrawal (2008) state the probability of regeneration decline with the increasing fuelwood needs from the forest commons. They found no evidence of the alleged negative association between the number of forest users and changes in forest condition [4].  Agrawal and Chhatre (2011), Lambin et al. (2001), and Rudel et al. (2002) used this variable to show its importance in explaining forest condition [5–7]. Agarwal and Chhatre (2011) found a negative association between the number of households and forest condition in their study sample [5]. |
| 1. Number of villages | A higher number of villages may indicate a higher probability of cooperation due to greater chances of representation from all segments of the society [8]. |
| 1. Number of farmers | More farmers are likely to result in more dependence on forests for meeting agricultural-based livelihood needs (fuelwood, fodder, grazing), which may lead to low vegetation growth[4]. |
| 1. Marginal population | We expect higher fuelwood dependence with increasing number of forest users belonging to marginalized sections of society. Chhatre and Agrawal (2008) state that the probability of regeneration declines with increasing fuelwood needs from forest commons. It is also an indicator of the poverty level of a village [4]. |
| 1. Number of literates | Agrawal and Chhatre (2011), Baland and Platteau (1996), Johnson and Forsyth (2002) used this variable (loosely related to EDUCATION) to show its importance in explaining forest condition [5,9,10].  Agrawal and Chhatre (2011, 2006) used this variable to show its importance in explaining forest condition. They show that higher levels of overall education positively relate to improved forest condition [5,11]. |
| 1. Unemployment | We expect higher unemployment as indicative of higher forest dependence. Unemployment is mainly off-farm, and in the absence of such off-farm opportunities, local villagers rely on farm income. Higher farm dependence may entail higher dependence on forestry resources for livestock and farm needs (fodder and fuelwood) [5]. |
| 1. Economic activity | Higher economic activity means the presence of alternative income opportunities. This may indicate higher off-farm income, therefore less reliance on farm income for livelihoods. Lower pressure on forests may result in improved forest condition [5]. |
| 1. Road density | Higher road density may result in more biotic pressure on forests resulting in poor long-term vegetation growth. It may also result in higher fire incidences due to wider usage of the forest landscape by humans who may negligently start fires by throwing cigarette butts after smoking or through other means (e.g. not dousing a campfire) in summer seasons. |
| 1. Number of small landholdings | Agrawal and Chhatre (2011) used a similar variable to show its importance in explaining forest condition. They mentioned it as “homogeneity,” which is the proportion of households with less than 0.4 hectares of agricultural land. They show that greater homogeneity positively relates to improved forest condition [5].  Chhatre (2007) found a higher number of large landholderings (> 2 hectares in the Himachal context) assists in overall cooperation, suggesting that a heterogeneity of endowments (within limits) is good for cooperation [8]. |
| **Subsystem 2: Governance System (GS) – Government rules and regulations** | |
| 1. Area planted | Chhatre and Agrawal (2008) found that in the presence of higher levels of enforcement, forests with improvement activities such as planting have > 50% probability of forest regeneration, even at a medium level of enforcement. They demonstrate that higher levels of enforcement relate to an increase in predicted probability of forest regeneration and a lower probability of forest degradation [4].  Agrawal and Chhatre (2011) used a PLANTATION variable to show its importance in explaining forest condition. They found that undertaking plantations in forest commons positively relate to forest condition outcomes [5]. |
| 1. Broadleaf spp. planted | Increasing the proportion of broadleaf species (BL) in a plantation mix suggests the utility of the species for the community; more stress on BL indicates more community needs for subsistence. More BL composition may create more community involvement and higher utility for forest users, suggesting higher enforcement and forest regeneration later on [5,12].  Agrawal and Chhatre (2011) found that subsistence use of forests (composition of forest in term of tree species: 1 = pure conifer, 2 = mixed, 3 = pure BL) relates positively with improved forest condition [5]. We also expect plantations that cater to subsistence needs of the people in terms of including a BL mix are more likely to succeed in the long term. |
| 1. Number of nurseries | Agrawal and Chhatre (2011) used a PLANTATION variable to show its importance in explaining forest condition [5]. Presence of nurseries, we expect, relates to the likelihood of plantations in forest beats. |
| **Subsystem 3: Resource Units (RU)** | |
| - Number of grazing animals | Agrawal and Chhatre (2011) and Tiffen et al. (1994) used this variable to show its importance in explaining forest condition [5,13].  Agrawal and Chhatre (2011) found grazing by goats and cattle to negatively influence forest condition [5]. |
| **Sub-system 4: Resource System (RS)** | |
| 1. Area of forest beat | Chhatre and Agrawal (2008) show that small- to medium-sized forest commons are associated with higher probability of regeneration. They found larger forests associated with a low probability of regeneration and a higher probability of degradation. However, in the presence of higher levels of enforcement, they demonstrate that even large forests have higher regeneration [4]. |
| 1. Tree cover | Agrawal and Chhatre (2011) found that previous forest condition positively associates with forest condition [5]. |
| 1. Crop acreage | Larger crop acreage in an FMR indicates the likelihood of local villagers being more dependent on forest resources. Higher dependence is likely to result in a low increase in vegetation outcomes. |
| 1. Grass acreage | Higher grass acreage in FMRs suggests presence of grazing-based livelihoods. It also means there are limits to spread of trees due to the nature of the grazing needs of the local and migratory livestock-raising communities. |
| 1. Bare land acreage | Higher bare land acreage may suggest more opportunities for extending tree cover, but it may also indicate limits to tree growth due to the presence of rocks, stones, and unproductive land. |
| 1. Soil depth | Deeper soils usually mean higher soil quality, which may assist long-term growth and establishment of planted saplings into trees, provided other social-ecological factors are supportive[14]. |
| 1. Total carbon | Presence of higher total carbon suggests more productive soils, which may facilitate long-term improvement in forest condition [14]. |
| 1. Total soil organic matter | Presence of higher amounts of soil organic matter suggests more productive soils, which may facilitate long-term improvement in forest condition [14]. |
| 1. Altitude | Higher elevations may limit tree growth due to the presence of rocks and low soil depth[14]. |
| 1. Available soil water capacity | Higher soil water capacity may suggest more productive soils, which may facilitate long-term improvement in forest condition. |
| 1. Baseline vegetation | Agrawal and Chhatre (2011) found that previous forest condition positively associates with forest condition [5]. We expect that higher vegetation levels in FMRs contribute to their positive growth outcome trajectories. |
| **Interactions (I) 🡺 Outcomes (O**) | |
| 1. Forest fire occurrences | Chhatre and Agrawal, 2008 found that the probability of deforestation and degradation declines with the increase in local enforcement (3). The occurrence of forest fires is an indicator of whether local enforcement is good or not. Forest fires are shown in the data to severely affect the likelihood of vegetation growth [4].  Agrawal and Chhatre (2011) found that the number of fires has a negative relation to forest condition [5]. |
| **Outcomes (O)** | |
| 1. Ecological performance measure (NDVI) | NDVI is used as proxy for estimating long-term vegetation growth[15]. |
| **Related ecosystems (ECO)** | |
| 1. ECO1 |  |
| 1. Temperature | Extreme temperatures limit tree growth[16–18]. |
| 1. Precipitation | Drier conditions hinder tree growth. |
| 1. Land surface temperature | Extreme temperatures limit tree growth due to excessively drier conditions[16–18]. |

**Table. D.** Trend in forest cover change since 1987 in Himachal Pradesh, India (in hectares) [19]

| **Year** | **1997** | **1999** | **2001** | **2003** | **2005** | **2007** | **2011** | **2013** | **2015** | **2017** |
| --- | --- | --- | --- | --- | --- | --- | --- | --- | --- | --- |
| Himachal Pradesh | 12,521 | 13,082 | 14,360 | 14,353 | 14,369 | 14,668 | 14,679 | 14,683 | 14,696 | 15,100 |
| Kangra District | 1,744 | 1,639 | 2,030 | 1,867 | 1,879 | 2,062 | 2,064 | 2,064 | 2,068 | 2,197 |

**Table. E.** Selected synthetically matched NDVI outcome trajectories (n = 30) for forest management regions (FMRs) in Kangra District, Himachal Pradesh, India, and their mean squared prediction error (LOSS V) and LOSS W values as calculated by nested optimization procedure for synthetic control matching. The shaded Jol and Kandi are not selected for analysis due to their nonplausible counterfactuals.

|  | **Random FMRs from 202 FMRs with plausible counterfactual trajectories** | **Mean squared prediction error (LOSS V)** | **LOSS W** |
| --- | --- | --- | --- |
| 1 | BALAKH | 0.00002 | 0.029 |
| 2 | BASSA | 0.0002 | 0.024 |
| 3 | BATUHI | 0.00002 | 0.007 |
| 4 | BINDRAWAN | 0.0002 | 0.03 |
| 5 | BORKA | 0.0001 | 0.11 |
| 6 | DAULATPUR | 0.0001 | 0.007 |
| 7 | FATEHPUR | 0.0001 | 0.06 |
| 8 | GHORAN | 0.00007 | 0.04 |
| 9 | GURIAL | 0.00005 | 0.005 |
| 10 | HAGWAL | 0.0002 | 0.02 |
| 11 | JAISINGHPUR | 0.00004 | 0.03 |
| 12 | JALARI | 0.00009 | 0.006 |
| 13 | JOL | 0.003 | 0.04 |
| 14 | KANDI | 0.0008 | 0.47 |
| 15 | KHABBAL | 0.00005 | 0.004 |
| 16 | KHANNI | 0.0001 | 0.02 |
| 17 | KHANPAT | 0.0001 | 0.13 |
| 18 | KOTHI | 0.00008 | 0.07 |
| 19 | LAHAT | 0.0002 | 0.01 |
| 20 | MAIRA | 0.0003 | 0.004 |
| 21 | MANGWAL | 0.00004 | 0.003 |
| 22 | NANGAL | 0.0001 | 0.01 |
| 23 | RAMEHAR | 0.00008 | 0.01 |
| 24 | REHLU | 0.00002 | 0.008 |
| 25 | RIRKMAR | 0.0003 | 0.04 |
| 26 | SALOL | 0.0001 | 0.01 |
| 27 | SATOBARI | 0.00008 | 0.01 |
| 28 | SOLDHA | 0.00005 | 0.08 |
| 29 | SUNHI | 0.00007 | 0.03 |
| 30 | TALARA | 0.0004 | 0.12 |

**Table. F.** Synthetic weights of SES indicators for 28 random forest management regions (FMRs) in Kangra District, Himachal Pradesh, India

**(a) FMRs 1–14 of 28**

| **Predictors** | **Balakh** | **Bassa** | **Batuhi** | **Bindrawan** | **Borka** | **Daulatpur** | **Fatehpur** | **Ghoran** | **Gurial** | **Hagwal** | **Jaisinghpur** | **Jalari** | **Khabbal** | **Khanni** |
| --- | --- | --- | --- | --- | --- | --- | --- | --- | --- | --- | --- | --- | --- | --- |
| Number of households | 0.03 | 0.05 | 0.01 | 0.03 | 0.04 | 0.19 | 0.23 | 0.02 | 0.24 | 0.00 | 0.04 | 0.05 | 0.04 | 0.04 |
| Number of villages | 0.01 | 0.05 | 0.04 | 0.04 | 0.02 | 0.02 | 0.00 | 0.00 | 0.01 | 0.04 | 0.03 | 0.00 | 0.03 | 0.00 |
| Number of farmers | 0.04 | 0.01 | 0.02 | 0.05 | 0.02 | 0.01 | 0.01 | 0.07 | 0.00 | 0.05 | 0.03 | 0.00 | 0.01 | 0.03 |
| Marginal population | 0.04 | 0.06 | 0.04 | 0.04 | 0.02 | 0.00 | 0.01 | 0.00 | 0.01 | 0.00 | 0.06 | 0.04 | 0.04 | 0.03 |
| Number of literates | 0.04 | 0.04 | 0.01 | 0.04 | 0.04 | 0.17 | 0.21 | 0.01 | 0.21 | 0.01 | 0.03 | 0.04 | 0.04 | 0.04 |
| Unemployment | 0.04 | 0.05 | 0.01 | 0.04 | 0.04 | 0.03 | 0.00 | 0.10 | 0.00 | 0.00 | 0.05 | 0.07 | 0.03 | 0.05 |
| Economic activity | 0.04 | 0.02 | 0.04 | 0.04 | 0.03 | 0.01 | 0.02 | 0.08 | 0.01 | 0.14 | 0.03 | 0.08 | 0.02 | 0.04 |
| Road density | 0.03 | 0.07 | 0.06 | 0.04 | 0.03 | 0.03 | 0.02 | 0.05 | 0.01 | 0.03 | 0.05 | 0.03 | 0.03 | 0.03 |
| Number of small landholdings | 0.04 | 0.04 | 0.04 | 0.05 | 0.03 | 0.04 | 0.01 | 0.04 | 0.00 | 0.02 | 0.03 | 0.04 | 0.05 | 0.04 |
| Area planted | 0.00 | 0.05 | 0.03 | 0.00 | 0.02 | 0.01 | 0.01 | 0.00 | 0.00 | 0.12 | 0.06 | 0.02 | 0.04 | 0.02 |
| Broadleaf sp. planted | 0.00 | 0.00 | 0.03 | 0.05 | 0.04 | 0.03 | 0.01 | 0.03 | 0.01 | 0.00 | 0.01 | 0.01 | 0.00 | 0.07 |
| Number of nurseries | 0.00 | 0.00 | 0.02 | 0.04 | 0.02 | 0.01 | 0.03 | 0.00 | 0.00 | 0.00 | 0.02 | 0.06 | 0.04 | 0.04 |
| Number of grazing animals | 0.03 | 0.05 | 0.05 | 0.04 | 0.02 | 0.02 | 0.00 | 0.07 | 0.00 | 0.00 | 0.04 | 0.00 | 0.01 | 0.01 |
| Forest beat area | 0.00 | 0.04 | 0.02 | 0.03 | 0.02 | 0.01 | 0.02 | 0.02 | 0.00 | 0.01 | 0.03 | 0.03 | 0.03 | 0.04 |
| Tree cover | 0.00 | 0.02 | 0.00 | 0.03 | 0.02 | 0.00 | 0.00 | 0.02 | 0.01 | 0.00 | 0.04 | 0.04 | 0.05 | 0.05 |
| Crop acreage | 0.06 | 0.02 | 0.01 | 0.04 | 0.02 | 0.02 | 0.01 | 0.10 | 0.01 | 0.01 | 0.02 | 0.03 | 0.03 | 0.04 |
| Grass acreage | 0.02 | 0.01 | 0.03 | 0.01 | 0.04 | 0.03 | 0.00 | 0.05 | 0.01 | 0.08 | 0.07 | 0.04 | 0.02 | 0.02 |
| Bare land acreage | 0.04 | 0.05 | 0.06 | 0.04 | 0.02 | 0.01 | 0.00 | 0.04 | 0.00 | 0.02 | 0.01 | 0.03 | 0.04 | 0.04 |
| Soil depth | 0.04 | 0.03 | 0.03 | 0.04 | 0.04 | 0.03 | 0.03 | 0.04 | 0.03 | 0.02 | 0.03 | 0.04 | 0.04 | 0.04 |
| Total carbon | 0.06 | 0.03 | 0.05 | 0.04 | 0.04 | 0.05 | 0.08 | 0.02 | 0.08 | 0.06 | 0.06 | 0.04 | 0.04 | 0.04 |
| Total organic carbon | 0.07 | 0.02 | 0.04 | 0.04 | 0.03 | 0.03 | 0.03 | 0.02 | 0.06 | 0.06 | 0.05 | 0.05 | 0.04 | 0.04 |
| Available soil water capacity | 0.07 | 0.04 | 0.03 | 0.03 | 0.02 | 0.03 | 0.03 | 0.03 | 0.04 | 0.04 | 0.02 | 0.03 | 0.04 | 0.04 |
| Baseline vegetation | 0.06 | 0.06 | 0.08 | 0.04 | 0.04 | 0.15 | 0.21 | 0.06 | 0.21 | 0.06 | 0.04 | 0.01 | 0.09 | 0.03 |
| Altitude | 0.04 | 0.03 | 0.05 | 0.04 | 0.04 | 0.03 | 0.02 | 0.04 | 0.01 | 0.07 | 0.04 | 0.04 | 0.06 | 0.04 |
| Forest fires | 0.07 | 0.04 | 0.06 | 0.04 | 0.02 | 0.02 | 0.02 | 0.01 | 0.00 | 0.06 | 0.01 | 0.02 | 0.02 | 0.02 |
| Temperature | 0.07 | 0.04 | 0.06 | 0.03 | 0.04 | 0.02 | 0.01 | 0.01 | 0.03 | 0.03 | 0.07 | 0.09 | 0.07 | 0.03 |
| Precipitation | 0.04 | 0.06 | 0.03 | 0.05 | 0.02 | 0.00 | 0.00 | 0.03 | 0.01 | 0.09 | 0.02 | 0.05 | 0.04 | 0.03 |
| Land surface temperature | 0.04 | 0.05 | 0.06 | 0.04 | 0.04 | 0.02 | 0.01 | 0.06 | 0.01 | 0.01 | 0.03 | 0.02 | 0.04 | 0.05 |

**(b) FMRs 14–28 of 28**

| **Predictors** | **Khanpat** | **Kothi** | **Lahat** | **Maira** | **Mangwal** | **Nangal** | **Ramehar** | **Rehlu** | **Rirkmar** | **Salol** | **Satobari** | **Soldha** | **Sunhi** | **Talara** |
| --- | --- | --- | --- | --- | --- | --- | --- | --- | --- | --- | --- | --- | --- | --- |
| Number of households | 0.02 | 0.04 | 0.04 | 0.23 | 0.25 | 0.04 | 0.04 | 0.25 | 0.24 | 0.03 | 0.24 | 0.04 | 0.04 | 0.01 |
| Number of villages | 0.02 | 0.04 | 0.04 | 0.01 | 0.01 | 0.00 | 0.04 | 0.01 | 0.00 | 0.02 | 0.00 | 0.00 | 0.02 | 0.00 |
| Number of farmers | 0.01 | 0.03 | 0.04 | 0.01 | 0.00 | 0.08 | 0.04 | 0.00 | 0.00 | 0.03 | 0.00 | 0.04 | 0.06 | 0.02 |
| Marginal population | 0.04 | 0.03 | 0.04 | 0.00 | 0.00 | 0.01 | 0.03 | 0.00 | 0.01 | 0.05 | 0.01 | 0.01 | 0.09 | 0.00 |
| Number of literates | 0.02 | 0.03 | 0.04 | 0.20 | 0.22 | 0.04 | 0.04 | 0.22 | 0.21 | 0.03 | 0.21 | 0.04 | 0.03 | 0.01 |
| Unemployment | 0.04 | 0.04 | 0.04 | 0.00 | 0.00 | 0.05 | 0.04 | 0.00 | 0.01 | 0.02 | 0.01 | 0.08 | 0.10 | 0.02 |
| Economic activity | 0.04 | 0.07 | 0.04 | 0.01 | 0.01 | 0.01 | 0.03 | 0.00 | 0.03 | 0.06 | 0.00 | 0.01 | 0.01 | 0.01 |
| Road density | 0.03 | 0.05 | 0.04 | 0.03 | 0.01 | 0.04 | 0.05 | 0.00 | 0.02 | 0.04 | 0.00 | 0.00 | 0.03 | 0.07 |
| Number of small landholdings | 0.11 | 0.03 | 0.04 | 0.01 | 0.01 | 0.02 | 0.03 | 0.01 | 0.01 | 0.02 | 0.01 | 0.02 | 0.04 | 0.00 |
| Area planted | 0.02 | 0.02 | 0.04 | 0.00 | 0.00 | 0.06 | 0.02 | 0.00 | 0.01 | 0.03 | 0.00 | 0.01 | 0.03 | 0.02 |
| Broadleaved sp. planted | 0.09 | 0.01 | 0.04 | 0.00 | 0.00 | 0.00 | 0.00 | 0.00 | 0.01 | 0.03 | 0.00 | 0.20 | 0.03 | 0.02 |
| Number of nurseries | 0.04 | 0.03 | 0.03 | 0.00 | 0.00 | 0.04 | 0.00 | 0.00 | 0.00 | 0.03 | 0.01 | 0.10 | 0.07 | 0.06 |
| Number of grazing animals | 0.05 | 0.03 | 0.04 | 0.01 | 0.00 | 0.00 | 0.04 | 0.01 | 0.01 | 0.03 | 0.00 | 0.03 | 0.04 | 0.00 |
| Forest beat area | 0.08 | 0.00 | 0.04 | 0.01 | 0.01 | 0.00 | 0.04 | 0.01 | 0.00 | 0.03 | 0.02 | 0.00 | 0.03 | 0.01 |
| Tree cover | 0.03 | 0.03 | 0.04 | 0.00 | 0.01 | 0.00 | 0.03 | 0.00 | 0.00 | 0.04 | 0.01 | 0.01 | 0.01 | 0.00 |
| Crop acreage | 0.01 | 0.03 | 0.04 | 0.00 | 0.01 | 0.05 | 0.05 | 0.01 | 0.03 | 0.03 | 0.01 | 0.03 | 0.01 | 0.06 |
| Grass acreage | 0.03 | 0.05 | 0.04 | 0.03 | 0.00 | 0.02 | 0.03 | 0.00 | 0.00 | 0.05 | 0.00 | 0.08 | 0.04 | 0.09 |
| Bare land acreage | 0.04 | 0.03 | 0.00 | 0.00 | 0.00 | 0.01 | 0.04 | 0.00 | 0.00 | 0.03 | 0.01 | 0.04 | 0.02 | 0.06 |
| Soil depth | 0.00 | 0.03 | 0.04 | 0.03 | 0.03 | 0.02 | 0.04 | 0.03 | 0.02 | 0.03 | 0.03 | 0.02 | 0.02 | 0.02 |
| Total carbon | 0.03 | 0.06 | 0.04 | 0.08 | 0.08 | 0.06 | 0.03 | 0.08 | 0.06 | 0.03 | 0.08 | 0.01 | 0.01 | 0.02 |
| Total organic carbon | 0.05 | 0.07 | 0.04 | 0.05 | 0.06 | 0.04 | 0.03 | 0.06 | 0.05 | 0.03 | 0.06 | 0.02 | 0.03 | 0.03 |
| Available soil water capacity | 0.00 | 0.06 | 0.04 | 0.04 | 0.04 | 0.01 | 0.03 | 0.04 | 0.01 | 0.03 | 0.04 | 0.01 | 0.04 | 0.03 |
| Baseline vegetation | 0.10 | 0.09 | 0.05 | 0.20 | 0.22 | 0.18 | 0.16 | 0.22 | 0.25 | 0.03 | 0.22 | 0.07 | 0.07 | 0.26 |
| Altitude | 0.00 | 0.03 | 0.00 | 0.02 | 0.01 | 0.05 | 0.04 | 0.02 | 0.00 | 0.08 | 0.01 | 0.05 | 0.02 | 0.05 |
| Forest fires | 0.02 | 0.00 | 0.04 | 0.00 | 0.01 | 0.04 | 0.03 | 0.00 | 0.01 | 0.03 | 0.00 | 0.00 | 0.02 | 0.01 |
| Temperature | 0.01 | 0.04 | 0.04 | 0.00 | 0.00 | 0.06 | 0.04 | 0.00 | 0.01 | 0.08 | 0.00 | 0.03 | 0.05 | 0.07 |
| Precipitation | 0.05 | 0.02 | 0.04 | 0.01 | 0.01 | 0.03 | 0.03 | 0.00 | 0.00 | 0.03 | 0.00 | 0.03 | 0.00 | 0.03 |
| Land surface temperature | 0.02 | 0.03 | 0.04 | 0.02 | 0.01 | 0.06 | 0.00 | 0.01 | 0.00 | 0.05 | 0.02 | 0.04 | 0.03 | 0.02 |

**Table. G.** Predictive synthetic control matching weights for social–ecological system (SES) indicators associated with normalized difference vegetation index (NDVI) increases in long-term vegetation growth, Kangra District, Himachal Pradesh, India

| **SES variables** | **Indicators** | **0%–10% increase in NDVI (low)** | **10%–20% increase in NDVI (medium)** | **> 20% increase in NDVI (high)** |
| --- | --- | --- | --- | --- |
| ***Actors*** | Number of households | 0.02 | **0.08** | **0.11** |
|  | Number of farmers | **0.07** | 0.02 | 0.03 |
|  | Number of marginal people | 0 | 0.03 | **0.04** |
|  | Number of villages | 0.002 | 0.02 | 0.02 |
|  | Number of literates | 0.01 | **0.08** | **0.10** |
|  | Number of unemployed people | **0.10** | 0.03 | **0.04** |
|  | Economic activity | **0.08** | 0.03 | 0.02 |
|  | Road density | **0.05** | 0.03 | 0.03 |
|  | Number of small landholdings | **0.04** | 0.03 | 0.03 |
| ***Governance System (planting program)*** | Forest area planted | 0 | 0.02 | 0.03 |
|  | Broadleaved sp. planted | 0.03 | 0.03 | 0.02 |
|  | Number of nurseries | 0 | 0.03 | 0.03 |
| ***Resource Units and Resource System*** | Number of grazing animals | **0.07** | 0.02 | 0.03 |
|  | Forest beat area | 0.02 | 0.02 | 0.03 |
|  | Tree cover | 0.02 | 0.02 | 0.02 |
|  | Crop acreage | **0.10** | 0.03 | 0.01 |
|  | Grass acreage | **0.05** | 0.03 | **0.04** |
|  | Bare land acreage | **0.04** | 0.03 | 0.02 |
|  | Soil depth | **0.04** | 0.03 | 0.03 |
|  | Total carbon | 0.02 | **0.05** | **0.05** |
|  | Total organic carbon | 0.02 | **0.04** | **0.04** |
|  | Altitude | **0.04** | 0.03 | 0.03 |
|  | Available soil water capacity | 0.03 | 0.03 | 0.03 |
|  | Baseline vegetation | **0.06** | **0.12** | **0.10** |
| ***Interactions*** | Number of forest fires | 0.01 | 0.02 | 0.02 |
| ***Related Ecosystems (climate factors)*** | Temperature | 0.01 | **0.04** | **0.04** |
|  | Land surface temperature | **0.06** | 0.03 | 0.03 |
|  | Precipitation | 0.03 | 0.03 | 0.01 |
| **Total predictive weights** | | 1.0 | 1.0 | 1.0 |
| **n** |  | 1 | 20 | 5 |

Note: Values of key predictors are in bold; two FMRs have experienced negative growth whose values are not given in the table.

**Table. H.** Direction of change in the values of indicators during the study period

| **SES subsystem** | **Indicator** | **Mean values of predictors^f^** | | **t-test**  **(p-value)** | **Plausible direction of influence^g^** |
| --- | --- | --- | --- | --- | --- |
|  |  | **Prior period mean** | **Subsequent period mean** |  |  |
| ***Actors*** |  |  |  |  |  |
| 1. Users | Number of households | 956 | 1187 | -1.98** (0.04) | +/- |
|  | Number of villages |  |  |  | -/+ |
|  | Number of farmers | 411 | 233 | 4.68*** (0.00) | - |
|  | Number of marginal people | 991 | 1132 | -1.15 (0.25) | - |
| 2. Socioeconomic conditions | Number of literates | 3284 | 4034 | -1.86* (0.06) | + |
|  | Number of unemployed people | 868 | 1135 | -2.69** (0.007) | - |
|  | Economic activity^a^ | 4.07 | 6.46 | -4.84*** (0.00) | + |
|  | Road density |  |  |  | -/+ |
| 3. Importance of resource | Number of small landholdings^b^ | 902 | 939 | -0.43 (0.67) | - |
| ***Governance System (planting program)*** | |  |  |  |  |
| 4. State afforestation programs | Forest area planted^c^ | 5.91 | 5.81 | 0.09 (0.92) | + |
|  | Broadleaf species planted^c^ | 90.19 | 90.62 | -0.14 (0.88) | + |
|  | Number of nurseries |  |  |  | + |
| ***Resource Units and Resource System*** | |  |  |  |  |
| 5. Mobile animals | Number of grazing animals^d^ | 5164.49 | 4776.44 | 1.01 (0.31) | - |
| 6. Size of resource system | Forest beat area |  |  |  | -/+ |
|  | Tree cover |  |  |  | + |
|  | Crop acreage |  |  |  | + |
|  | Grass acreage |  |  |  | + |
|  | Bare land acreage |  |  |  | +/- |
| 7. System productivity | Soil depth |  |  |  | + |
|  | Total carbon |  |  |  | + |
|  | Total organic carbon |  |  |  | + |
|  | Available soil water capacity |  |  |  | + |
|  | Baseline vegetation (NDVI) | 0.48 | 0.51 | -4.67*** (0.00) | + |
| 8. Location | Altitude |  |  |  | - |
| ***Interactions*** |  |  |  |  |  |
| 9. Conflicts among users | Number of forest fires^e^ | 0.05 | 0.09 | -1.31 (0.19) | - |
| ***Related Ecosystems (climatic factors)*** | |  |  |  |  |
| 10. Climatic factors | Temperature | 18.26 | 18.22 | 0.09 (0.78) | + |
|  | Precipitation | 76.69 | 77.45 | -0.47 (0.64) | +/- |
|  | Land surface temperature | 297.14 | 296.91 | 0.62 (0.54) | + |
| Total predictive weights | |  |  |  |  |

^a^ Nighttime Lights data 2002–2008 is prior period and Nighttime Lights data 2009–2015 is subsequent period

^b^ Agricultural census 2005 is prior period and agricultural census 2011 is subsequent period

^c^ Plantation 2002–2008 is prior period and 2009-2015 is subsequent period

^d^ Livestock census 2007 is prior period and agricultural census 2012 is subsequent period

^e^ Fire data 2002–2008 is prior period and 2009-2015 is subsequent period

^f^ For variables for which data are available for prior and subsequent periods; Census 2001 is prior period and Census 2011 is subsequent period

^g^ For indicative references, see Table S2.

Note:*, **, *** significant at 10%, 5%, 1%, respectively;

**Table. I.** Synthetically matched NDVI outcome trajectories for forest management regions (FMRs) in Kangra District, Himachal Pradesh, India, 2002-2016.

|  | **Random FMRs from 202 FMRs with plausible counterfactual trajectories** | Baseline  Mean  Annual  NDVI  (2002) | Mean Annual  NDVI (2016) | Mean Annual NDVI  Change  (yr2016- yr2002) | Baseline literacy  (number of  literates, 2002) | Literacy, 2011  (number of  literates, 2011) | Literacy  Change  (number of literates, yr2011- yr2002) | Baseline Farmer Population  (2002) | Farmer Population  (2011) | Change in Number of Farmers (yr2011-  yr2002) |
| --- | --- | --- | --- | --- | --- | --- | --- | --- | --- | --- |
| 1 | BALAKH | 0.45 | 0.5 | 0.06 | 1576 | 1910 | 334 | 407 | 131 | -276 |
| 2 | BASSA | 0.48 | 0.56 | 0.07 | 1719 | 2173 | 454 | 193 | 87 | -106 |
| 3 | BATUHI | 0.47 | 0.55 | 0.08 | 577 | 722 | 145 | 94 | 62 | -32 |
| 4 | BINDRAWAN | 0.48 | 0.55 | 0.07 | 2643 | 3563 | 920 | 298 | 203 | -95 |
| 5 | BORKA | 0.45 | 0.54 | 0.09 | 563 | 693 | 130 | 216 | 41 | -175 |
| 6 | DAULATPUR | 0.45 | 0.56 | 0.11 | 2882 | 3590 | 708 | 591 | 18 | -573 |
| 7 | FATEHPUR | 0.43 | 0.49 | 0.05 | 6387 | 8343 | 1956 | 681 | 689 | 8 |
| 8 | GHORAN | 0.45 | 0.47 | 0.02 | 3171 | 3813 | 642 | 410 | 676 | 266 |
| 9 | GURIAL | 0.46 | 0.52 | 0.06 | 8981 | 12019 | 3038 | 1041 | 611 | -430 |
| 10 | HAGWAL | 0.44 | 0.36 | -0.08 | 847 | 1358 | 511 | 26 | 92 | 66 |
| 11 | JAISINGHPUR | 0.47 | 0.59 | 0.12 | 6314 | 7165 | 851 | 847 | 66 | -781 |
| 12 | JALARI | 0.44 | 0.53 | 0.09 | 4018 | 4780 | 762 | 794 | 422 | -372 |
| 13 | JOL | 0.5 | 0.56 | 0.06 | 707 | 1117 | 410 | 30 | 152 | 122 |
| 14 | KANDI | 0.45 | 0.46 | 0.01 | 1882 | 1882 | 0 | 18 | 18 | 0 |
| 15 | KHABBAL | 0.46 | 0.52 | 0.06 | 870 | 907 | 37 | 251 | 9 | -242 |
| 16 | KHANNI | 0.41 | 0.48 | 0.07 | 2912 | 3566 | 654 | 308 | 281 | -27 |
| 17 | KHANPAT | 0.47 | 0.55 | 0.08 | 1636 | 1872 | 236 | 412 | 13 | -399 |
| 18 | KOTHI | 0.42 | 0.48 | 0.06 | 446 | 564 | 118 | 73 | 302 | 229 |
| 19 | LAHAT | 0.51 | 0.6 | 0.09 | 3441 | 3825 | 384 | 339 | 21 | -318 |
| 20 | MAIRA | 0.42 | 0.5 | 0.07 | 265 | 375 | 110 | 27 | 54 | 27 |
| 21 | MANGWAL | 0.46 | 0.45 | -0.02 | 1484 | 1850 | 366 | 238 | 405 | 167 |
| 22 | NANGAL | 0.41 | 0.49 | 0.07 | 1710 | 2314 | 604 | 248 | 298 | 50 |
| 23 | RAMEHAR | 0.52 | 0.61 | 0.09 | 6240 | 7297 | 1057 | 1075 | 490 | -585 |
| 24 | REHLU | 0.48 | 0.56 | 0.07 | 5650 | 7153 | 1503 | 537 | 119 | -418 |
| 25 | RIRKMAR | 0.43 | 0.51 | 0.08 | 915 | 1104 | 189 | 166 | 16 | -150 |
| 26 | SALOL | 0.45 | 0.52 | 0.07 | 479 | 639 | 160 | 8 | 42 | 34 |
| 27 | SATOBARI | 0.44 | 0.55 | 0.11 | 1412 | 2445 | 1033 | 28 | 33 | 5 |
| 28 | SOLDHA | 0.45 | 0.54 | 0.09 | 1549 | 1757 | 208 | 445 | 97 | -348 |
| 29 | SUNHI | 0.44 | 0.57 | 0.13 | 3379 | 3888 | 509 | 574 | 127 | -447 |
| 30 | TALARA | 0.51 | 0.57 | 0.06 | 123 | 132 | 9 | 35 | 13 | -22 |

Note: n = 30. Shaded FMRs (Jol and Kandi) were not included in the analysis due to their nonplausible counterfactuals.

**References**

1. Ostrom E. A general framework for analyzing sustainability of social-ecological systems. Science. 2009;325: 419–422.

2. Chen J, Chen J, Liao A, Cao X, Chen L, Chen X, et al. Global land cover mapping at 30 m resolution: A POK-based operational approach. ISPRS J Photogramm Remote Sens. 2015;103: 7–27.

3. Wieder WR, Boehnert J, Bonan GB, Langseth M. Regridded harmonized world soil database v1. 2. Data set. Available -Line Httpdaac Ornl Gov Oak Ridge Natl Lab Distrib Act Arch Cent Oak Ridge Tenn USA. 2014;

4. Chhatre A, Agrawal A. Forest commons and local enforcement. Proc Natl Acad Sci. 2008;105: 13286–13291.

5. Agrawal A, Chhatre A. Strengthening causal inference through qualitative analysis of regression residuals: explaining forest governance in the Indian Himalaya. Environ Plan A. 2011;43: 328–346.

6. Lambin EF, Turner BL, Geist HJ, Agbola SB, Angelsen A, Bruce JW, et al. The causes of land-use and land-cover change: moving beyond the myths. Glob Environ Change. 2001;11: 261–269.

7. Rudel TK, Bates D, Machinguiashi R. A tropical forest transition? Agricultural change, out-migration, and secondary forests in the Ecuadorian Amazon. Ann Assoc Am Geogr. 2002;92: 87–102.

8. Chhatre A. Democracy on the commons: Political competition and local cooperation for natural resource management in India. PhD Thesis, Duke University. 2007.

9. Baland J-M, Platteau J-P. Halting degradation of natural resources: is there a role for rural communities? Food & Agriculture Org.; 1996.

10. Johnson C, Forsyth T. In the eyes of the state: Negotiating a “rights-based approach” to forest conservation in Thailand. World Dev. 2002;30: 1591–1605.

11. Agrawal A, Chhatre A. Explaining success on the commons: Community forest governance in the Indian Himalaya. World Dev. 2006;34: 149–166.

12. Poteete AR, Ostrom E. Heterogeneity, group size and collective action: the role of institutions in forest management. Dev Change. 2004;35: 435–461.

13. Tiffen M, Mortimore M, Gichuki F. More people, less erosion: environmental recovery in Kenya. John Wiley & Sons Ltd; 1994.

14. OADES JM. Soil organic matter and structural stability: mechanisms and implications for management. : 19.

15. Pettorelli N, Vik JO, Mysterud A, Gaillard J-M, Tucker CJ, Stenseth NC. Using the satellite-derived NDVI to assess ecological responses to environmental change. Trends Ecol Evol. 2005;20: 503–510.

16. Went FW. The effect of temperature on plant growth. Annu Rev Plant Physiol. 1953;4: 347–362.

17. Hatfield JL, Prueger JH. Temperature extremes: effect on plant growth and development. Weather Clim Extrem. 2015;10: 4–10.

18. Körner C. Paradigm shift in plant growth control. Curr Opin Plant Biol. 2015;25: 107–114.

19. FSI. Forest Survey of India, Dehradun, India [Internet]. [cited 18 Jul 2018]. Available: http://fsi.nic.in/index.php
